# Regulation of PCGEM1 by p54/nrb in prostate cancer

**DOI:** 10.1038/srep34529

**Published:** 2016-09-29

**Authors:** Tsui-Ting Ho, Jianguo Huang, Nanjiang Zhou, Ziqiang Zhang, Pratirodh Koirala, Xinchun Zhou, Fangting Wu, Xianfeng Ding, Yin-Yuan Mo

**Affiliations:** 1 Department of Pharmacology and Toxicology, Cancer Institute, University of Mississippi Medical Center, Jackson, MS, USA; 2 Department of Radiation Oncology, University of Mississippi Medical Center, Jackson, MS, USA; 3 Department of Biochemistry, Cancer Institute, University of Mississippi Medical Center, Jackson, MS, USA; 4 Department of Radiation Oncology, Duke University Medical Center, Durham, NC, USA; 5 System Biosciences, Mountain View, CA, USA; 6 Department of Pulmonary Medicine, Tongji Hospital, Tongji University, Shanghai, China; 7 Department of Pathology, University of Mississippi Medical Center, Jackson, MS, USA; 8 College of Life Sciences, Zhejiang Sci-Tech University, Hangzhou, China

## Abstract

PCGEM1 is a long non-coding RNA (lncRNA) that is often upregulated in prostate cancer. However, little is known how PCGEM1 is regulated. In the present study, we show transcriptional regulation of PCGEM1 in response to androgen deprivation by p54/nrb. While ectopic expression of p54/nrb increases, suppression of p54/nrb by RNAi or knockout (KO) reduces PCGEM1. Moreover, rescue experiments indicate that re-expression of p54/nrb in KO cells restores the ability to induce PCGEM1, leading to upregulation of the androgen receptor splice variant AR3 which has been shown to play a role in castration resistance. Finally, 3,3′-Diindolylmethane (DIM), a known chemoprevention agent, is capable of suppressing PCGEM1 expression by preventing the interaction of p54/nrb with the PCGEM1 promoter. In particular, DIM reduces tumor growth by suppression of PCGEM1 and promoting apoptosis in the castrated xenograft mouse model. Together, these results demonstrate a novel mechanism of p54/nrb-mediated expression of PCGEM1 and AR3, contributing to castration resistance in prostate cancer.

Prostate cancer (PCa) is the second leading cause of death in men in the USA^1^. The androgen receptor (AR) is required for prostate development and prostate cancer pathogenesis. Thus, AR serves as a principal therapeutic target, and androgen deprivation therapy (ADT) has been the mainline treatment for aggressive PCa^2^,^3^. Despite the high initial response rates, these tumors ultimately develop the resistance, i.e., castration-resistant prostate cancer (CRPC)^4^. It is widely viewed as a key obstacle to successful therapy; however, the underlying molecular mechanism(s) for the emergence of CRPC is not fully understood. Recent studies have demonstrated that constitutive expression of AR splice variants lacking the ligand binding domain significantly contribute to the development and progression of CRPC^5^. Among these, the most studied AR splice variant, AR-V7 or AR3, activates AR regulated genes in the absence of ligands, and therefore could play a critical role in castration resistance^6^. For instance, it has been recently shown that circulating AR3 is associated with the resistance to two clinically important drugs enzalutamide and abiraterone^7^.

Long non-coding RNAs (lnRNAs) are arbitrarily defined as a group of non-coding RNAs with molecular weight larger than 200 nucleotides in length^8^. Although lncRNAs are poorly characterized, increasing evidence suggests their important regulatory roles in regulation of diverse cellular processes. Dysregulation of lncRNAs is often associated with numerous diseases, including PCa^9^. Prostate cancer gene expression marker 1 (PCGEM1) is one of the early identified oncogenic lncRNAs^10^. Upregulation of PCGEM1 has been associated with high risk of PCa^11^ and ectopic expression of PCGEM1 contributes to resistance to doxorubicin-induced apoptosis^12^. A long serial analysis of gene expression (long SAGE) library suggested its role in PCa castration recurrent stage^13^. Furthermore, PCGEM1 has been implicated in the activation of the AR transcription activity in CRPC^14^. However, despite being discovered nearly 15 years ago, little literature information is available regarding transcriptional regulation of PCGEM1.

P54/nrb is a 55 kDa ubiquitously expressed protein originally identified as a non-POU domain-containing, octamer binding protein^15^. It plays various roles in the nucleus, including transcriptional regulation, RNA splicing, nuclear retention, and subnuclear body formation^16^. Moreover, its expression, localization, and interactions with transcription factors have been implicated in cancers^17^,^18^. However, it remains to be determined as to whether p54/nrb plays any role in regulation of lncRNAs such as PCGEM1.

In the present study, we show that p54/nrb is a positive regulator of PCGEM1, contributing to AR3 expression and castration resistance.

## Results

### Upregulation of PCGEM1 is correlated with AR3 expression and castration resistance

**Figure 1:**
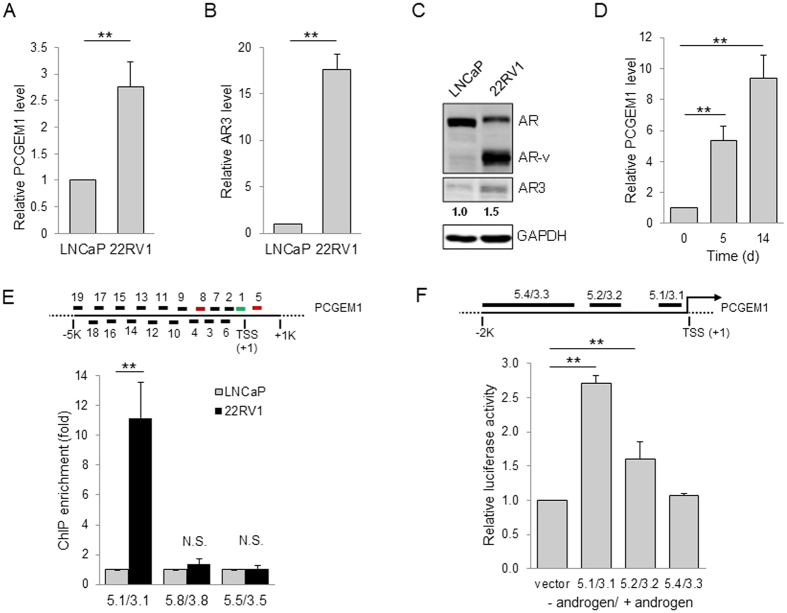
Transcriptional regulation of PCGEM1 in castration resistance. Detection of (**A**) PCGEM1 and (**B**) AR3 in LNCaP and CWR22Rv1 (22RV1) cells by qRT-PCR. (**C**) Relative expression of AR, AR variants (AR-v) and AR3 in LNCaP and CWR22Rv1 cells, as detected by Western using N-terminal AR antibody or AR3 specific antibody. (**D**) AD induces PCGEM1 in LNCaP cells. The cells were cultured in the absence of androgen for up to 14 days before harvesting to qRT-PCR. (**E**) (Top) Schematic presentation of PCGEM1 promoter, and the location of the respective primers used for PCR analysis. The numbers below the PCGEM1 promoter indicate the position relative to TSS. (Bottom) Histone ChIP assays to confirm an active binding site using primer sets highlighted in color. (**F**) (Top) Schematic presentation of PCGEM1 promoter, and the location of the respective primer sequences for PCR cloning. The numbers below the PCGEM1 promoter indicate the position relative to TSS. (Bottom) Induction of the PCGEM1 promoter luciferase activity. Different sizes of DNA fragments upstream of PCGEM1 were cloned into pGL3-Basic as a luciferase reporter. LNCaP cells were transfected with the luciferase reporter and cultured in the absence or presence of androgen for 3 days before harvesting for luciferase assay. Values are mean ± SE (n = 3). **p < 0.01; N.S., not significant. Full-length gels and blots are included in the Supplementary Information file.

We previously showed that PCGEM1 contributes to castration resistance by regulating expression of AR3^19^, and thus, the primary goal of this study was to determine how PCGEM1 is regulated in PCa to impact castration resistance. To this end, we examined PCGEM1 expression in LNCaP and CWR22Rv1 cells because LNCaP is androgen sensitive whereas CWR22Rv1 is androgen insensitive. In consistent with our previous findings, the PCGEM1 level was much higher in CWR22Rv1 cells than that in LNCaP cells (Fig. 1A). AR3 was also higher in CWR22Rv1 cells than in LNCaP cells at both mRNA and protein levels (Fig. 1B,C). In addition, we found that PCGEM1 was progressively induced in LNCaP and LNCaP95 cells by androgen-deprivation (AD) (Fig. 1D; Fig. S1).

### Transcriptional regulation of PCGEM1

Since active promoters are marked by trimethylation of Lys4 of histone H3 (H3K4me3)^20^, and it is the methylation state associated with transcriptional start sites of actively transcribed genes^21^, we carried out ChIP assays and qPCR to assess H3K4 trimethylation within a ∼5 kb region upstream of PCGEM1 transcription start site (TSS). We identified the active transcription of PCGEM1 within the first 1 kb fragment (Fig. 1E, top). For example, there was over an 11-fold enrichment of PCGEM1 by H3K4me3 antibody over IgG for 5.1/3.1 region, but neither in distal upstream region (5.8/3.8) nor in the downstream (5.5/3.5) in the CWR22Rv1 cells compared to LNCaP cells (Fig. 1E, bottom); androgen deprivation increases the binding of H3K4 to PCGEM1 promoter in LNCaP cells (Fig. S2), suggesting a transcriptional activation of PCGEM1. To test this possibility, we constructed reporter vectors containing the putative PCGEM1 promoter sequences. Consistent with histone ChIP results, a high level of luciferase activity was detected within the upstream region close to the TSS (~500 bp) (Fig. 1F) in LNCaP cells in response to AD. By contrast, no activation was observed in far upstream, suggesting that this 500 bp region is critical for its transcriptional activation.

### p54/nrb as a potential regulator for PCGEM1

**Figure 2:**
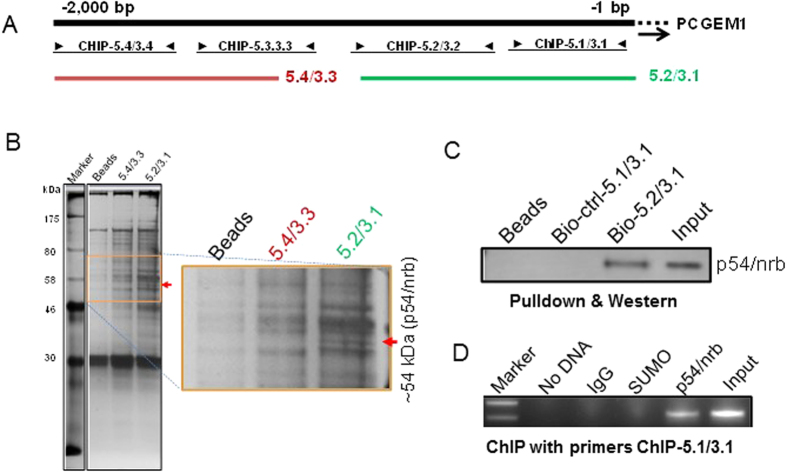
p54/nrb is a potential regulator for PCGEM1 expression. (**A**) Schematic presentation of PCGEM1 promoter, and the location of the respective PCR primers for pulldown assays. The numbers above the PCGEM1 indicate the position relative to TSS. (**B**) A representative silver-stained gel picture showing a band unique to a biotin-labeled probe 5.2/3.1. Mass spectrometry analysis indicated that this band is Non-POU domain-containing octamer-binding protein (NONO), also known as p54/nrb. (**C**) Verification of the interaction of PCGEM1 with p54/nrb by p54/nrb antibody for the same precipitates as in (**B**). (**D**) The interaction of p54/nrb with PCGEM1 promoter by ChIP assays using primers PCGEM1-ChIP-5.1 and PCGEM1-ChIP-3.1 in 22Rv1 cells. Full-length gels and blots are included in the Supplementary Information file.

To search for potential factors involved in the regulation of PCGEM1, we performed pulldown assays using DNA fragments illustrated in Fig. 2A. SDS-PAGE analysis of precipitate, followed by silver staining, revealed a unique band at ~54 kDa to the probe 5.2/3.1 (Fig. 2B, right); mass spectrometry analysis suggested Non-POU domain-containing octamer-binding protein (NONO), also known as p54/nrb, as a potential factor for PCGEM1 regulation. Pulldown with biotinylated PCGEM1 probes and Western blot using p54/nrb antibody confirmed their interaction (Fig. 2C). Finally, ChIP assays with p54/nrb antibody revealed that p54/nrb specifically interacted with the PCGEM1 promoter (Fig. 2D).

**Figure 3:**
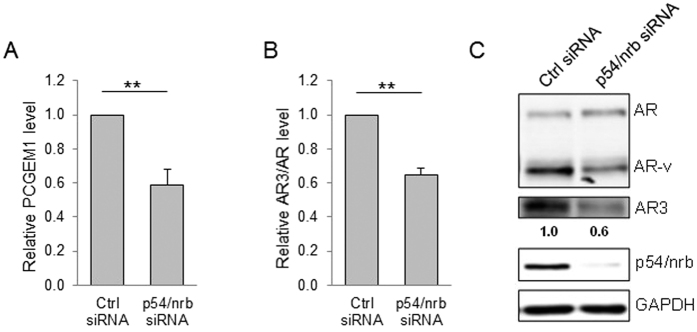
p54/nrb is required for PCGEM1 expression. (**A**) Suppression of the PCGEM1 by p54/nrb-siRNA as detected by qRT-PCR. Suppression of the AR3 by p54/nrb-siRNA as detected by (**B**) qRT-PCR and (**C**) Western using N-terminal AR antibody or AR3 specific antibody. Values are mean ± SE (n = 3). **p < 0.01. Full-length gels and blots are included in the Supplementary Information file.

To determine the function of this interaction, we suppressed p54/nrb by RNAi and demonstrated that p54/nrb siRNA significantly reduced PCGEM1 (Fig. 3A). At the same time, p54/nrb siRNA was able to suppress AR3 at the mRNA and protein level in CWR22Rv1 cells (Fig. 3B,C), further supporting that p54/nrb is involved in the regulation of PCGEM1.

### Regulation of PCGEM1 and AR3 expression by p54/nrb

**Figure 4:**
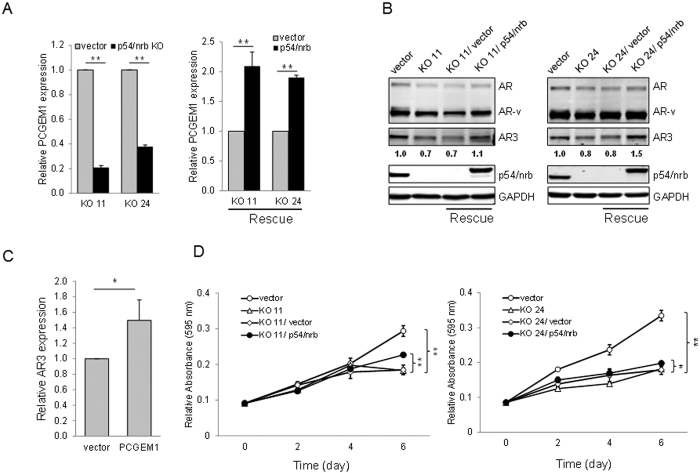
Re-expression of p54/nrb in the knockout cells restores PCGEM1 and AR3 expression. (**A**) Two independent CWR22Rv1 p54/nrb knockout (KO) clones #11 and #24 were used for rescue experiments. Re-expression of p54/nrb significantly increases the PCGEM1 level. (**B**) Re-expression of p54/nrb significantly increases the AR3 level, as determined by Western blot. (**C**) Ectopic expression of PCGEM1 in p54/nrb KO #11 cells increases AR3 as detected by qRT-PCR. The exogenous p54/nrb was tagged with Myc. (**D**) Re-expression of p54/nrb enhances tumor cell growth. KO #11 and #24 cells were transduced with pCDH-Myc vector (vector) or pCDH-Myc-p54/nrb (p54/nrb) and then plated out in 96-well plates in the absence of androgen. Relative cell growth was measured by MTT assays at Day 6. Values are mean ± SE (n = 3). **p < 0.01; *p < 0.05. Full-length gels and blots are included in the Supplementary Information file.

To further determine the role of p54/nrb in the regulation of PCGEM1, we knocked out p54/nrb by CRISPR/Cas9 technology using a dual gRNA approach (Fig. S3) as described previously^22^. Initial characterization by genomic PCR identified several knockout (KO) clones, which were then verified by qRT-PCR and Western blot. We selected KO clones #11 and #24 for further characterization. As expected, p54/nrb KO substantially suppressed PCGEM1 expression in both clones (Fig. 4A, left). Importantly, re-expression of p54/nrb in the KO cells (rescue) significantly increased PCGEM1 expression (Fig. 4A, right). Furthermore, p54/nrb KO caused a reduction of AR3 (Fig. 4B). Similarly, re-expression of p54/nrb increased AR3 expression. Ectopic expression of PCGEM1 in p54/nrb KO cells also increased AR3 expression (Fig. 4C). We then asked whether the increased expression of PCGEM1 and AR3 by rescue experiments confers castration resistance. MTT and nuclear staining assays revealed that the suppression of cell growth in the KO cells was partially rescued by re-expression of p54/nrb (Fig. 4D; Fig. S4). However, ectopic expression of p54/nrb had no effect on PCGEM1 promoter activity (Fig. S5) presumably because the level of endogenous p54/nrb is high. Together, these results suggest that PCGEM1 is functionally regulated by p54/nrb.

### Synergistic activation mediator (SAM)-mediated activation of PCGEM1 increases AR3 expression

To further determine the role of PCGEM1 in regulation of AR3, we used engineered Cas9 SAM (Synergistic Activation of Mediator) system^23^. Unlike traditional ectopic expression of a given gene, this approach is able to activate the corresponding endogenous gene. Thus, we designed 5 single-guide RNAs (sgRNAs) targeting the proximal PCGEM1 promoter between −1 kb and the +1 TSS (Fig. S6A). Introduction of a mixture of five sgRNAs in LNCaP cells caused PCGEM1 activation (Fig. S6B), and upregulation of AR3 (Fig. S6C). SAM sgRNAs had little effect on AR (Fig. S6D). Overall, upregulation of PCGEM1 by SAM caused an increase ratio of AR3/AR (Fig. S6E).

### The binding of p54/nrb to the promoter region is critical for the p54/nrb-mediated regulation of PCGEM1

**Figure 5:**
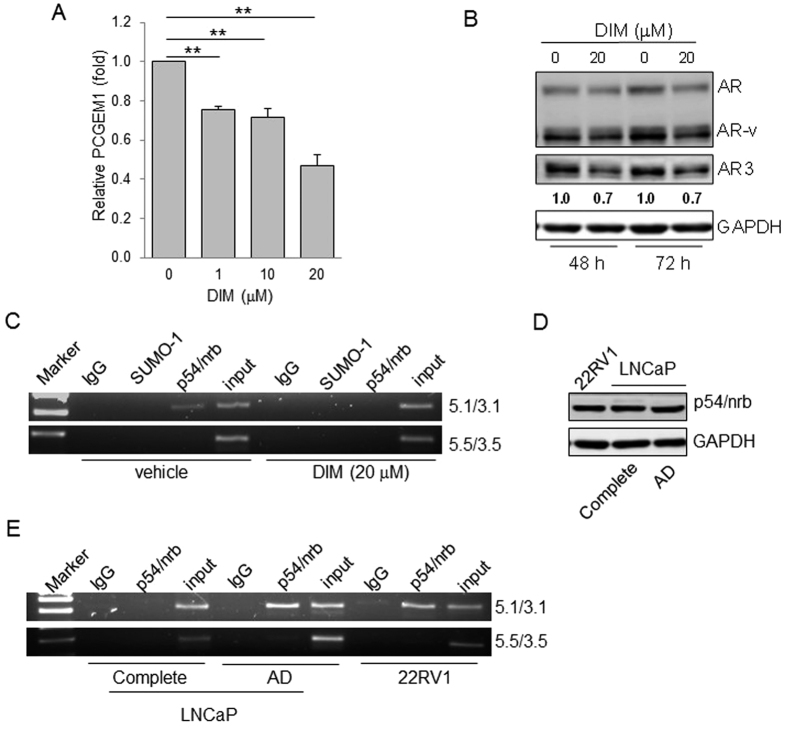
DIM decreases PCGEM1 expression by preventing p54/nrb from binding to PCGEM1 promoter. (**A**) CWR22Rv1 cells were treated with vehicle control (DMSO) or the indicated concentrations of DIM for 24 h, followed by qRT-PCR analysis. (**B**) CWR22Rv1 cells were treated with vehicle control (DMSO) or 20 μM of DIM for 48 h and 72 h, and the AR, AR-v, and AR3 protein levels were determined by Western blot. (**C**) Binding of p54/nrb to the PCGEM1 promoter in CWR22Rv1 cells, as detected by ChIP assays. ChIP assays with CWR22Rv1 cells under 20 μM of DIM for 3 days were performed using p54/nrb antibody, and the precipitated chromatins were PCR-amplified with the use of specific primers (PCGEM1-ChIP, 5.1/3.1 and PCGEM1-ChIP, 5.5/3.5). SUMO antibody serves as a negative control. (**D**) Relative expression of p54/nrb in CWR22Rv1, LNCaP cells in the presence (complete) or absence of androgen (androgen-deprivation; AD) for 7 days, as detected by Western blot. (**E**) Androgen deprivation promotes the binding of p54/nrb with the PCGEM1 promoter. ChIP assays with LNCaP cells in the presence or absence of androgen (AD) for 3 days were performed using p54/nrb antibody, and the precipitated chromatins were PCR-amplified with the use of specific primers (PCGEM1-ChIP-5.1/3.1 and PCGEM1-ChIP-5.5/3.5). CWR22Rv1 cells serve as a control. Values are mean ± SE (n = 3). **p < 0.01. Full-length gels and blots are included in the Supplementary Information file.

3,3′-Diindolylmethane (DIM) is a natural compound found in cruciferous vegetables. It has been recently shown that DIM can suppresses prostate tumor growth possibly by downregulation of AR^24^, however, little is known with regard to its role in PCGEM1 expression. We showed that DIM downregulated PCGEM1 expression in a dose-dependent manner (Fig. 5A). Similarly, DIM caused a decrease in the expression of AR3 (Fig. 5B). Therefore, we used DIM as a research tool to dissect the mechanism of PCGEM1 regulation by p54/nrb. We first determined the effect of DIM on p54/nrb expression and found that DIM had no effect on the p54/nrb level (Fig. S7). To determine whether DIM suppresses PCGEM1 through p54/nrb, we performed ChIP assays with p54/nrb antibody. Of great interest, the interaction of p54/nrb with PCGEM1 promoter (5.1/3.1) was abolished in the presence of DIM (Fig. 5C), suggesting that DIM prevents p54/nrb from binding to the promoter region, leading to suppression of PCGEM1. Similarly, AD was able to induce PCGEM1 in LNCaP cells (Fig. 1D), but it had no effect on p54/nrb expression (Fig. 5D). Moreover, ChIP assays with p54/nrb antibody detected the interaction of p54/nrb with PCGEM1 promoter in the AD treated LNCaP cells whereas no such interaction was seen in LNCaP cells grown in regular medium (Fig. 5E). In consistent with a high level of PCGEM1 in CWR22Rv1 cells (Fig. 1A), we also detected the interaction between p54/nrb and the PCGEM1 promoter, similar to what was seen in the AD treated LNCaP cells. These results suggest that the increased interaction of p54/nrb with the PCGEM1 promoter contributes to the increased expression of PCGEM1 in LNCaP cells under AD and constitutive upregulation of PCGEM1 in CWR22Rv1 cells.

### DIM suppresses the PCGEM1-mediated castration resistance

**Figure 6:**
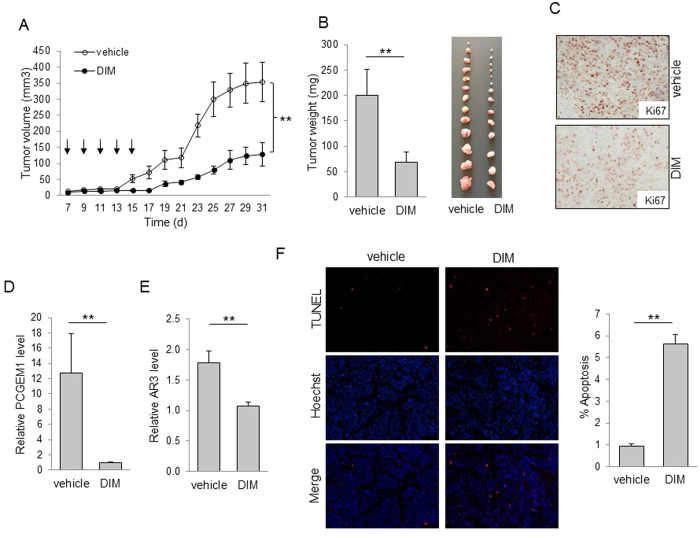
DIM reduces prostate tumor growth by suppression of PCGEM1 in the castrated xenograft mouse model. (**A**) Effect of DIM on tumor growth in a xenograft mouse model. Male SCID mice were first castrated and one week later, CWR22Rv1 cells were injected into flanks as described in the materials and methods. Arrows indicate DIM administration. Tumor growth was measured every other day after 7 days of injection. (**B**) Tumors were harvested at day 31 and weighed. Actual tumor size after harvest was shown in the right panel. (**C**) DIM suppresses Ki-67 signal. The excised tumors were formalin-fixed, paraffin-embedded, and stained with Ki-67 antibody. (**D**) DIM suppresses PCGEM1 and (**E**) AR3 in xenograft tumors. RNA was extracted from three individual tumors. (**F**) DIM induces apoptosis in tumors as determined by TUNEL assays. Apoptotic cells were counted in 10 random fields per group under a fluorescent microscope (×20 magnification). Values in D and E are mean ± SE (n = 3). **p < 0.01.

**Figure 7:**
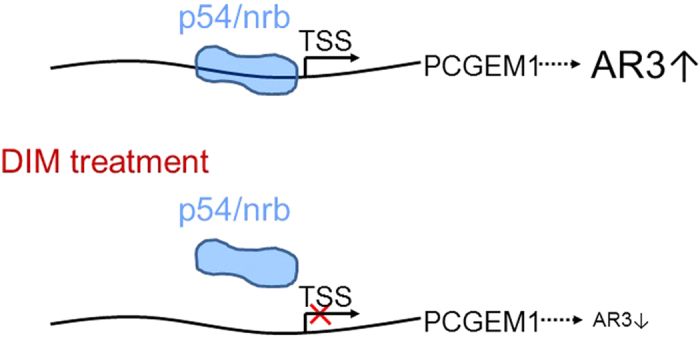
A proposed model for p54/nrb-mediated regulation of PCGEM1. Once cells are treated with DIM, DIM prevents p54/nrb from binding to PCGEM1 promoter, and decreases the expression of PCGEM1, thus resulting in the down-regulation of AR3. TSS: transcription start site.

We then determined whether downregulation of PCGEM1 by DIM can suppress castration resistance. MTT assays demonstrated that DIM sensitized LNCaP and CWR22Rv1 cells to AD (Fig. S8). Of interest, suppression of PCGEM1 by DIM significantly reduced tumor growth (Fig. 6A) and the tumor weight in castrated male mice (Fig. 6B). Moreover, we detected a stronger Ki-67 signal in tumors derived from control group than DIM treated tumors (Fig. 6C). These results suggest that the decreased PCGEM1 by DIM might contribute to suppression of tumor growth. We also showed that DIM can reduce PCGEM1 and AR3 in the castrated xenograft mouse model, as determined by qRT-PCR (Fig. 6D,E). Finally, TUNEL assays indicated that DIM increased tumor cell apoptosis. For example, the number of apoptotic cells was about 5.6-fold higher in DIM treated tumors than in control tumors (Fig. 6F). Similar results were observed in the cell culture after DIM treatment, supporting that DIM can increase apoptosis both *in vitro* and *in vivo*. Together, these results suggest that DIM-related tumor growth suppression is associated with the downregulation of PCGEM1 and AR3 possibly through its interaction with PCGEM1 promoter (Fig. 7).

## Discussion

Although the prognosis for prostate cancer is in general favorable, still more than 32,000 U.S. men die of metastatic disease annually^4^. This is largely attributed to the development of castration resistance. It is well known that multiple factors have been implicated in castration resistance; expression of constitutively active AR splice variants plays a significant role. Our previous work suggests a role for PCGEM1 in regulation of AR splice variants such as AR3^19^. In the present study, we provide further evidence that androgen deprivation induces PCGEM1 through p54/nrb, leading to expression of AR3 and castration resistance.

P54/nrb is a multi-functional nuclear protein involved in a variety of nuclear processes^25^. Especially, p54/nrb plays a role in RNA splicing and gene regulation^26^. Regarding gene regulation, p54/nrb is well known to regulate hormone receptor signaling. For instance, p54/nrb is necessary for glucocorticoid induction of occludin and claudin-5 expression, and has been implicated in normal blood-retinal barrier induction *in vivo*^27^. Of interest, p54/nrb may function as an activator or repressor. In one case, p54/nrb serves as a transcriptional corepressor of progesterone receptor (PR)^28^. In addition, p54/nrb along with PSF suppresses AR transcriptional activity, which can be reversed by the inhibition of histone deacetylase activity^29^. In another case, p54/nrb has been shown to interact with AR in a ligand-dependent manner, thus functioning as a coactivator of AR to potentiate transcription^30^. Finally, p54/nrb plays an important role in cAMP-dependent activation of CREB target^31^. Therefore, the identification of p54/nrb as a PCGEM1 transcriptional regulator in this study further expands the repertoire of its targeted genes including both coding genes and lncRNAs.

As a transcriptional regulator, p54/nrb may provoke DNA-binding proteins to form a functional complex or enhance repressor activity to inhibit transcriptional activity^28^,^30^. P54/nrb has been shown to regulate gene expression by binding to the unmethylated promoter^32^, enhancer region^33^, or independence of direct contact with nucleic acids^34^. Until now there is no information available as to whether p54/nrb can regulate expression of lncRNAs. Our work provides a first piece of evidence that p54/nrb is a positive regulator of PCGEM1, by physically interacting with the PCGEM1 promoter. Knockdown of p54/nrb by RNAi or KO by CRISPR/Cas9 causes a significant reduction of the PCGEM1 level; on the other hand, re-expression of p54/nrb in the KO cells increases the PCGEM1 level.

Although androgen deprivation induces PCGEM1 expression, it has no effect on the p54/nrb. In consistent with this finding, while the PCGEM1 level is higher in castration resistant CWR22Rv1 cells than in androgen sensitive LNCaP cells, there is no difference for p54/nrb in these two cell lines. Moreover, DIM is capable of suppressing PCGEM1, but has no effect on the p54/nrb level. Instead, the interaction of p54/nrb with PCGEM1 promoter is critical to PCGEM1 expression. For instance, DIM significantly reduces the binding of p54/nrb to the PCGEM1 promoter (Fig. 5C); in contrast, androgen deprivation increases this binding (Fig. 5E). Hence, in addition to being a chemopreventive or therapeutic agent, DIM provides a research tool for dissection of p54/nrb-mediated regulation of PCGEM1.

The detailed mechanism of p54/nrb-mediated PCGEM1 expression still remains to be determined. At least two possibilities exist. One possibility is that the interaction of p54/nrb with the PCGEM1 promoter may make the local DNA structure more accessible to specific binding proteins or basic transcription machinery^34^. Another possibility is related to the primary structure of p54/nrb. It is known that p54/nrb has stretches of glutamines and a proline-rich region in its N-terminus and C-terminus, respectively, and these domains have been implicated in protein-protein interactions^35^. Through interaction of these putative domains with DNA-binding proteins, p54/nrb might also facilitate the formation of protein-DNA complexes, leading to activation of transcription.

DIM belongs to a member of naturally occurring plant alkaloids and is found in abundance in cruciferous vegetables such as broccoli^36^. It is a potent agent for the inhibition of prostate cancer cell growth through multiple cellular signaling pathways^37^. Of interest, DIM is also implicated in the regulation of AR signaling^24^. For example, BR-DIM (formulated DIM) treatment downregulates the expression of AR variants and AR3 in CWR22Rv1 cells and suppresses the formation of prostaspheres derived from CWR22Rv1 cells^37^. Moreover, a phase II clinical trial (NCT00888654) has been conducted to assess the value of BR-DIM in the treatment of PCa^38^. Although this might involve expression of microRNAs, the role of DIM on lncRNA expression is not clear. Thus, our study provides an additional molecular explanation for suppression of castration resistance by DIM.

The number of lncRNAs is increasing rapidly, driven primarily by advanced technology such as next-generation RNA sequencing and bioinformatics analysis tools^39^,^40^. Since lncRNAs are often transcribed by RNA polymerase II, their expression can be regulated by well-established transcription factors such as p53 or Myc^41^,^42^ or in a tissue-specific manner^43^. In addition, genetic aberrations (deletions and amplifications)^44^,^45^ or epigenetic alterations (DNA methylation or histone modification)^46^,^47^ may be attributed to abnormal lncRNA expression. We herein present evidence that PCGEM1 can be regulated through p54/nrb in response to androgen deprivation. Thus, ADT is a double edge sword. On one hand, it kills prostate tumor cells; on the other hand, it also induces PCGEM1 such that these surviving cells become castration resistant. Accordingly, interruption of ADT-induced PCGEM1 expression may help to overcome castration resistance.

## Materials and Methods

### Reagents

Primary antibody against full-length AR (D6F11; #5153) was purchased from Cell Signaling Technology Inc. (Danvers, MA); AR3 (AG10008) from Precision Antibody (Columbia, MD); SUMO-1 (D-11; sc-5308) and p54/nrb (E3; sc-376806) from Santa Cruz Biotechnology (Dallas, TX); Histone H3K4me3 (Catalog No.39159) from Active Motif (Carlsbad, CA); GAPDH (Clone:1E6D9) from ProteinTech (Chicago, IL). Secondary antibodies conjugated with IRDye 800CW or IRDye 680 were purchased from LI-COR Biosciences (Lincoln, NE). Pooled siRNAs against p54/nrb and control siRNA were purchased from Santa Cruz Biotechnology. PCR primers, biotin-labeled PCGEM1 probes and control oligos were purchased from IDT (Coralville, IA). 3,3′-Diindolylmethane (DIM) was purchased from Sigma-Aldrich (St. Louis, MO).

### Cell culture

Prostate cancer LNCaP and CWR22Rv1 cells were obtained from ATCC (Manassas, VA); Cells were cultured in RPMI-1640 (Lonza, Walkersville, MD) with 10% FBS (Sigma-Aldrich, St. Louis). Cells under androgen-deprivation were grown in phenol free RPMI 1640 supplemented with charcoal stripped 5% FBS (Sigma-Aldrich). All media contained 2-mM glutamine, 100 units of penicillin/ml and 100 mg of streptomycin/ml. Cells were incubated at 37 °C and supplemented with 5% CO_2_ in a humidified chamber. LNCaP and CWR22Rv1 cells were authenticated by DDC Medical (http://www.ddcmedical.com) using the short tandem repeat profiling method.

### Construction of plasmids

PCR reactions for cloning purpose used high fidelity enzyme Phusion (New England BioLabs, Ipswich, MA). Expression vector for p54/nrb was pCDH-EF1-MCS-GFP-T2A-PU from SBI (CD550A-1); Cas9-dual gRNA vector was previously described^22^ and construction of p54/nrb dual gRNA (Supplementary Table 1) followed the same strategy as previously described^22^. Donor vector was constructed by firstly amplifying left and right arms from the human genomic DNA and then sequentially cloned into the donor vector carrying GFP-PU^22^. For luciferase assays, respective fragments upstream of PCGEM1 transcription start site were separately cloned into pGL3-basic vector at Kpn I and Xho I sites. All PCR products were verified by DNA sequencing.

### Knockout of p54/nrb by CRISPR/Cas9

Selection of p54/nrb knockout clones in CWR22Rv1 cells was similar to what had been described previously^22^. Briefly, p54/nrb dual gRNA and donor vector were co-transfected into CWR22Rv1 cells. One week later, puromycin was added 1 μg/ml to cell culture and were further grown for 2 weeks. The surviving cells were subject to cell sorting by FACS based on GFP signal into 96-well plates, followed by expansion in 12-well plates. Initial identification was carried out by genomic PCR. Potential clones were further verified by qRT-PCR and Western blot.

### Design and cloning of SAM sgRNAs

We designed 5 sgRNAs to target 1 KB upstream of PCGEM1 from transcription start site (TSS) using CHOPCHOP program^48^ (https://chopchop.rc.fas.harvard.edu/). Self-complementary oligos were ligated into the BsmB1 site of lenti sgRNA (MS2)_zeo backbone^23^, a gift from Feng Zhang (Addgene plasmid # 61426).

### RNA isolation, RT-PCR and qRT-PCR

We isolated total RNA using Direct-zol^TM^ RNA MiniPrep (Zymo Research, Irvine, CA) per the manufacturer’s protocol and used 0.5 μg RNA to synthesize cDNA by RevertAid Reverse Transcriptase (Fisher Scientific, Pittsburgh, PA) with random primer mix (New England BioLabs). The resultant cDNA was used for PCR reactions. PCR annealing temperature varied depending on the primers used. To specifically detect expression of PCGEM1, full-length AR, AR3, we used the SYBR Green method with primers described previously^19^. β-actin or GADPH was used as an internal control. Delta-delta Ct values were used to determine their relative expression as fold changes, as previously described^22^.

### Western blot

Cells were harvested and protein was extracted from cells as previously described^49^. The protein concentration was determined using Bio-Rad Protein Assay Dye Reagent (Bio-Rad, Hercules, CA) and samples were separated in sodium dodecyl sulfate polyacrylamide gels.

### Chromatin immunoprecipitation Assays

Chromatin immunoprecipitation (ChIP) assays were performed using EZ-ChIP^TM^ (Millipore, Billerica, MA) according to the manufacturer’s protocol. Briefly, cells were first fixed with 1% formaldehyde, and chromatin DNA was isolated and bound protein was digested with proteinase K. PCR was performed using primers PCGEM1-ChIP-5.1 and PCGEM1-ChIP-3.1 or control primers PCGEM1-ChIP-5.5 and PCGEM1-ChIP-3.5/ PCGEM1-ChIP-5.8 and PCGEM1-ChIP-3.8 (Supplementary Table 1). IgG and an unrelated antibody (anti-SUMO) were used as negative controls.

### Transfection

Cells were transfected with siRNAs using RNAfectin reagent (Applied Biological Materials, Richmond, BC, Canada) or plasmid DNA using DNAfectin (Applied Biological Materials) following the manufacturer’s protocol.

### Luciferase assays

Luciferase assays were performed using Dual-Luciferase^TM^ Reporter Assay System (Promega, Madison, WI) according to the manufacturer’s protocol. Briefly, LNCaP cells were first transfected with appropriate plasmids in 12-well plates, and then cultured in the absence or presence of androgen. Three days after transfection, the cells were harvested and lysed for luciferase assays. Renilla luciferase was used for normalization.

### Protein ID identification by mass spectrometry

PCGEM1 promoter DNA probes used for precipitation assays were prepared by standard PCR using biotin-labeled primers. After precipitation, samples were separated in SDS-PAGE, followed by silver staining using Pierce^TM^ Silver Stain Kit (Fisher Scientific) as previously described^50^. Candidate protein bands were carefully cut out and sent out for mass spectrometry analysis provided by Applied Biomics (Hayward, CA).

### Immunohistochemistry

Immunohistochemistry (IHC) was used to detect Ki-67 in xenograft tumors using UltraVision^TM^ ONE Detection System (Fisher Scientific) per the manufacturer’s protocol as previously described^51^.

### TUNEL (terminal deoxynucleotidyl transferase dUTP nick end labeling) assay

TUNEL assays were performed in both *in situ* CWR22Rv1 cells from xenograft and *in vitro* CWR22Rv1 cells treated with vehicle or DIM using *In Situ* Cell Death Detection Kit, TMR red (Roche, Indianapolis, IN) per the manufacturer’s protocol. The rest of the procedure has been previously described^52^.

### MTT assay

MTT assay was performed to determine the effect of DIM in cell growth in response to androgen deprivation. 2000 (complete medium) or 5000 (androgen-deprivation medium) LNCaP cells per well were seeded in triplicate into 96-well plates and grown in the presence or absence of 50 μM of DIM or CWR22Rv1. The measurement was carried out 4 days after DIM treatment. MTT assay was also performed to determine the effect of p54/nrb in cell growth in response to androgen deprivation. Two thousands of CWR22Rv1 cells per well were seeded in triplicate into 96-well plates and the measurement was carried out for 0~6 days after seeding.

### Xenograft mouse model

The animal studies were conducted in accordance with NIH animal use guidelines and the experimental protocol approved by the UMMC’s Animal Care and Use Committee. Male SCID mice at 5~6 week old purchased from Charles River (Wilmington, MA) were first castrated, and one week later CWR22Rv1 cells were injected subcutaneously into these mice with 1 million cells containing 50% matrigel per spot. One week after injection, mice were randomly grouped to receive intraperitoneal injection of 20 mg/kg DIM or vehicle (corn oil) every other day for 10 days. Tumor growth was monitored every other day and harvested at day 31 after injection. Tumor volumes were calculated as: (π/6) × [length (mm) × width^2^ (mm^2^)].

### Statistical Analysis

Comparisons between groups were analyzed using the Student’s *t*-test (two groups) or a one-way ANOVA followed by post hoc Tukey test (multiple groups). Differences with *P* values less than 0.05 are considered significant.

## Additional Information

**How to cite this article**: Ho, T.-T. *et al.* Regulation of PCGEM1 by p54/nrb in prostate cancer. *Sci. Rep.* **6**, 34529; doi: 10.1038/srep34529 (2016).

## Supplementary Material

Supplementary Information
