# Supplementary material for: Regulation of PCGEM1 by p54/nrb in prostate cancer
Source: Sci Rep. 2016 Sep 29;6:34529. doi: 10.1038/srep34529 (PMC5041109; doi:10.1038/srep34529)
Supplement: Supplementary Information [file srep34529-s1.pdf]

Regulation of PCGEM1 by p54/nrb in prostate cancer

Tsui-Ting Ho[1], Jianguo Huang[2, 3], Nanjiang Zhou[2,4], Ziqiang Zhang[5], Pratirodh Koirala[2], Xinchun Zhou[6], Fangting Wu[4], Xianfeng Ding[1,7], Yin-Yuan Mo[1*]

Supplementary Table 1, Primers used in this study

ChIP Primers

PCGEM1-ChIP-5.1	ttgatcttagcatgcttcac
PCGEM1-ChIP-5.2	gaaaaaattgctgggttata
PCGEM1-ChIP-5.3	gaaaacatgcatatgtttcc
PCGEM1-ChIP-5.4	tttaaagtttcaggttgttg
PCGEM1-ChIP-3.1	caggagcactgagggatatt
PCGEM1-ChIP-3.2	caagacataaagtagaaagt
PCGEM1-ChIP-3.3	agcacaaataatataactac
PCGEM1-ChIP-3.4	aatggactcatacaaacatc
PCGEM1-ChIP-5.5	tttagtgtaattagcaggga
PCGEM1-ChIP-3.5	gaaactgtgtgccctgaaaa
PCGEM1-ChIP-5.6	ctttatgtcttgtgatggat
PCGEM1-ChIP-3.6	tacaacgtgcagtgaagcct
PCGEM1-ChIP-5.7	atctctttgacattcgacta
PCGEM1-ChIP-3.7	tgatttggcattatcttgta
PCGEM1-ChIP-5.8	aaagtgatatgagcactgga
PCGEM1-ChIP-3.8	gatgatgactttatgggtgg
PCGEM1-ChIP-5.9	catgagtttcaagctagaga
PCGEM1-ChIP-3.9	gtgttagtggcagtttagca
PCGEM1-ChIP-5.10	attctgtgcttattatccag
PCGEM1-ChIP-3.10	tcacagactaagagatgggt
PCGEM1-ChIP-5.11	gcatctcagtattaagtgga
PCGEM1-ChIP-3.11	acagccaactgatacaacaa
PCGEM1-ChIP-5.12	atctgcattcttcattaaac
PCGEM1-ChIP-3.12	gctatctaaatctaaggtta
PCGEM1-ChIP-5.13	gtatgggcaattcttgctca
PCGEM1-ChIP-3.13	gaagaatgcagatttcaacc
PCGEM1-ChIP-5.14	aggaatacagagcaccgact
PCGEM1-ChIP-3.14	gcccatacaagccctaatcc
PCGEM1-ChIP-5.15	agattggtcccttatatcaa
PCGEM1-ChIP-3.15	cctaggatttaggatttgta
PCGEM1-ChIP-5.16	agcgtcaccaaagagatagt
PCGEM1-ChIP-3.16	ggaccaatctcctgtttact
PCGEM1-ChIP-5.17	gcagaaccaactttaaagat
PCGEM1-ChIP-3.17	tgtagccacaatagctctaa
PCGEM1-ChIP-5.18	tttgttaaagctgatttaga
PCGEM1-ChIP-3.18	ttgtatgtgtgatacctaga
PCGEM1-ChIP-5.19	caagacaacactgaaggatt
PCGEM1-ChIP-3.19	aataatgactgaagagctgc

To make SAM sgRNAs against the PCGEM1 promoter

PCGEM1p-5.1A	CACCgTGGCAGAACAAGCAACTATG
PCGEM1p-5.2A	CACCgGGGCACCAATATACTCATAC
PCGEM1p-5.3A	CACCgTGCTCCCCATTTTTTCCTAT
PCGEM1p-5.4A	CACCgTGAATTAACAGGAGCACTGA
PCGEM1p-5.5A	CACCgTGAAATTATGTATAAAACAG
PCGEM1p-3.1B	aaacCATAGTTGCTTGTTCTGCCAc

PCGEM1p-3.2B	aaacGTATGAGTATATTGGTGCCCc
PCGEM1p-3.3B	aaacATAGGAAAAAATGGGGAGCAc
PCGEM1p-3.4B	aaacTCAGTGCTCCTGTTAATTCAc
PCGEM1p-3.5B	aaacCTGTTTTATACATAATTTCAc

To clone p54/nrb

NONO-Myc-R1-5.1	CCATGGAGGCCCGAATTCTGCAGAGTAATAAAACTTTTAA
NONO-Myc-Not1--3.1	TCGCAGATCCTTGCGGCCGCTTAGTATCGGCGACGTTTGT

To knock out p54/nrb

NONO gRNA1	AGTGCTCTGGCTTTTGTCAG
NONO gRNA2	TGAGGCATGTGCTCTAAAGG
NONO-left-recomb-5.1	CCTCCAGCTCCAGCCTCCTG
NONO-left-recomb-3.1	TTTGTTACTTTGAACAACCA
NONO-right-recomb-5.1	AGCTGGAGAATAATAGATGT
NONO-right-recomb-3.1	TGCCTGGTTAATTCAATTTA

To make PCGEM1 luciferase reporters

Luc-B-PCGEM1p-Kpn-5.1	TTTCTCTATCGATAGGTACCTTGATCTTAGCATGCTTCAC
Luc-B-PCGEM1p-Xho1-3.1	CTTAGATCGCAGATCTCGAGCAGGAGCACTGAGGGATATT
Luc-B-PCGEM1p-Kpn-5.2	TTTCTCTATCGATAGGTACCGAAAAAATTGCTGGGTTATA
Luc-B-PCGEM1p-Xho1-3.2	CTTAGATCGCAGATCTCGAGCAAGACATAAAGTAGAAAGT
Luc-B-PCGEM1p-Kpn-5.4	TTTCTCTATCGATAGGTACCTTTAAAGTTTCAGGTTGTT
Luc-B-PCGEM1p-Xho1-3.3	CTTAGATCGCAGATCTCGAGAGCACAAATAATATAACTAC

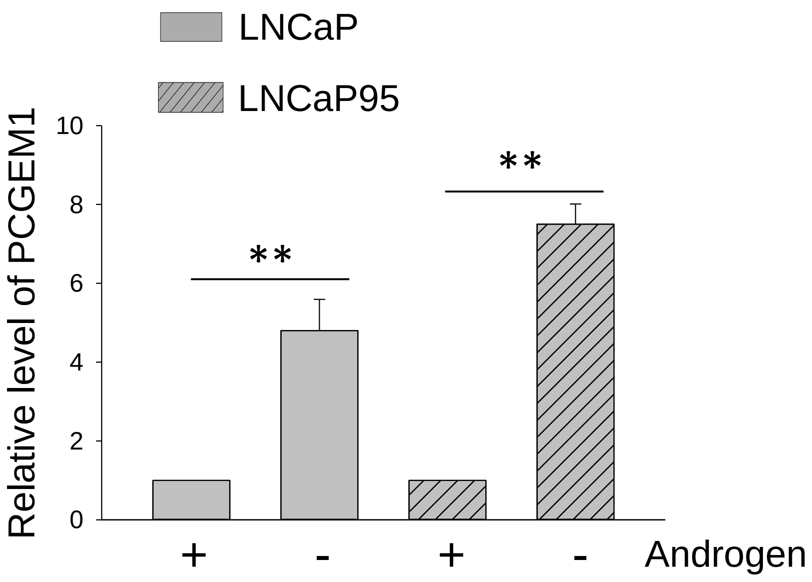

Fig. S1 Induction of PCGEM1 by androgen deprivation. Detection of PCGEM1 in LNCaP and LNCaP95, which is an androgen-independent cell line derived from long-term continuous culture of LNCaP cells in androgen-depleted conditions by qRT-PCR. PCGEM1 was progressively induced in LNCaP and LNCaP95 cells by androgen-deprivation (- androgen).

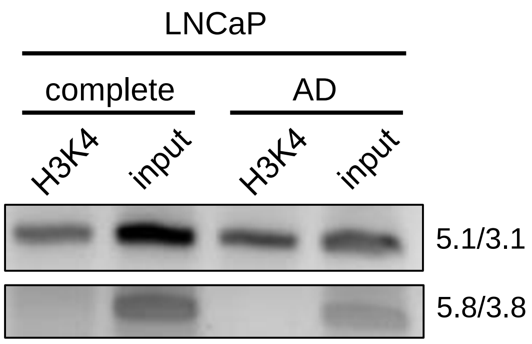

Fig. S2 Androgen deprivation increases the binding of H3K4 to PCGEM1 promoter in LNCaP cells, as determined by histone ChIP assays.

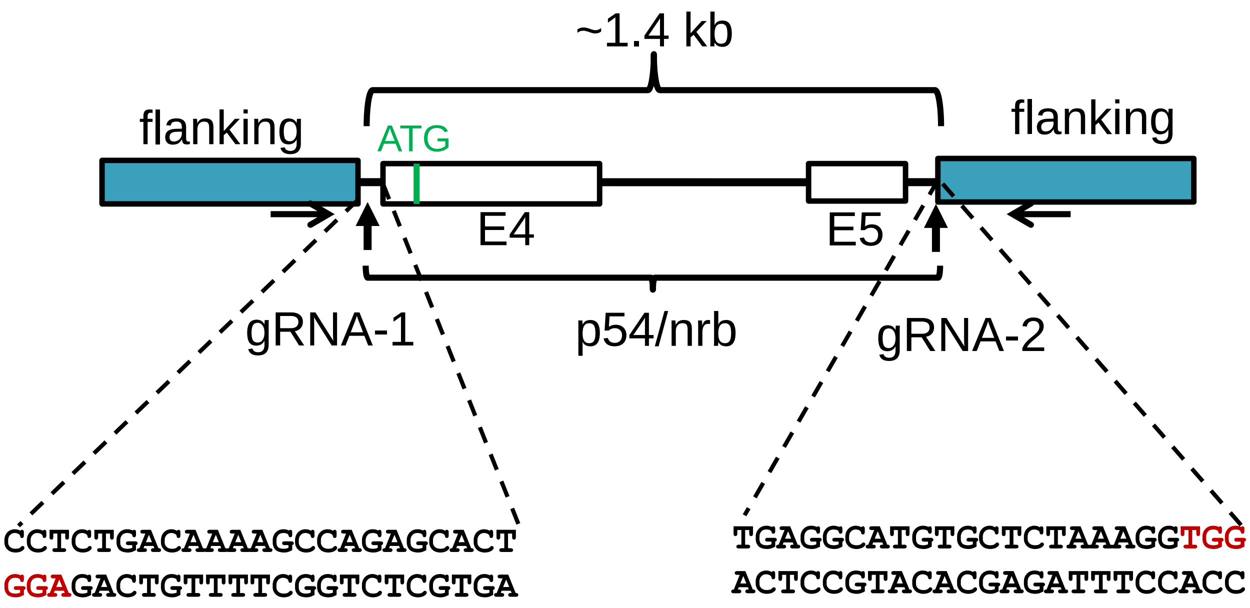

Fig. S3 Strategy for knockout of p54/nrb in CWR22Rv1 cells.

Ho, Fig. S4

A

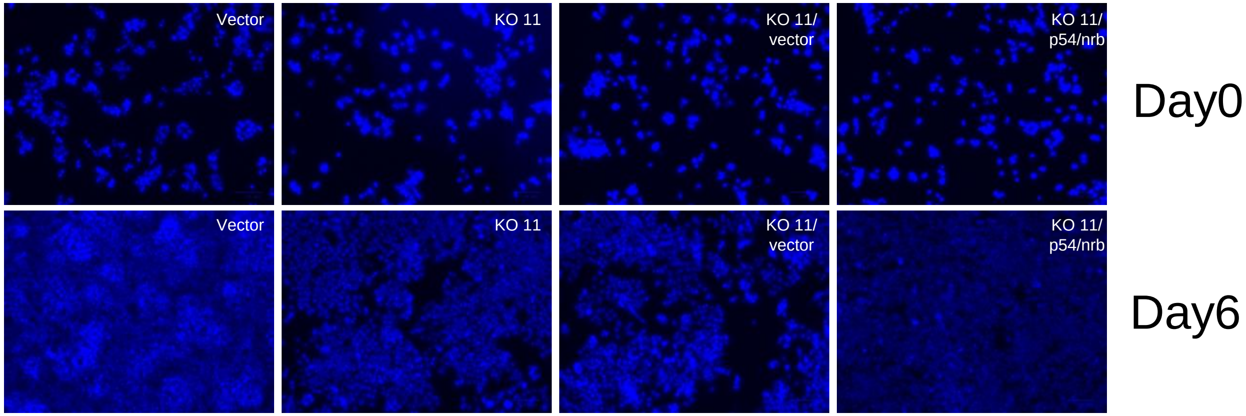

B

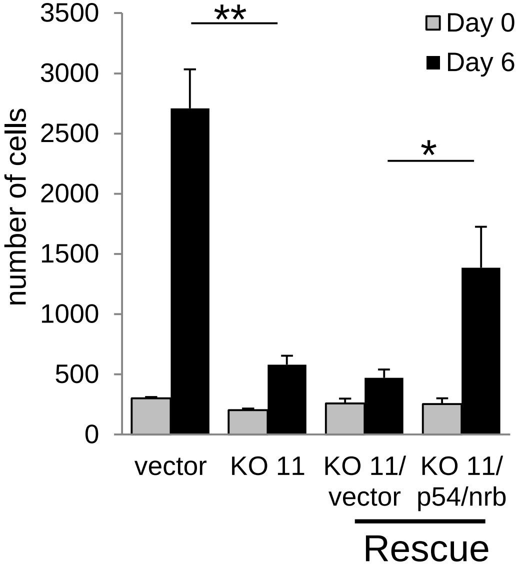

Fig. S4 Re-expression of p54/nrb enhances tumor cell growth. (A) p54/nrb KO #11 cells were transduced with pCDH-Myc vector (vector) or pCDH-Myc-p54/nrb (p54/nrb) and then plated out in 12-well plates in the absence of androgen. Relative cell growth was measured by hoechst staining at Day 6. Numbers of cells were counted in 3 random fields per group under a microscope (×20 magnification). (B) Quantification of hoechst staining data. Values are mean ± SE (n = 3). *, $p < 0.05$**; $p < 0.01$.

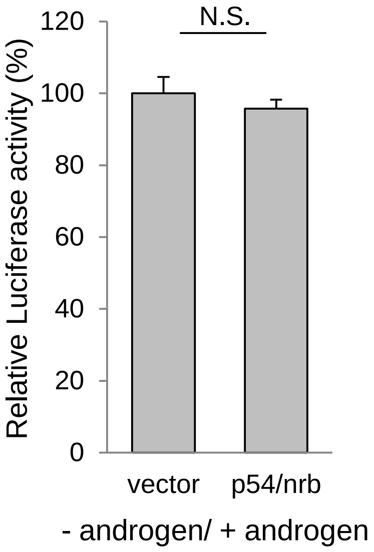

Fig. S5. Ectopic expression of p54/nrb has no effect on PCGEM1 promoter luciferase activity. 5.1/3.1 fragment upstream of PCGEM1 was cloned into pGL3-Basic as a luciferase reporter (refer to Fig 1F, top). LNCaP cells were co-transfected with the luciferase reporter and either vector or p54/nrb, and cultured in the presence or absence of androgen (- androgen or + androgen) for 3 days before harvesting for luciferase assay. Values are mean ± SE (n = 3). N.S., not significant.

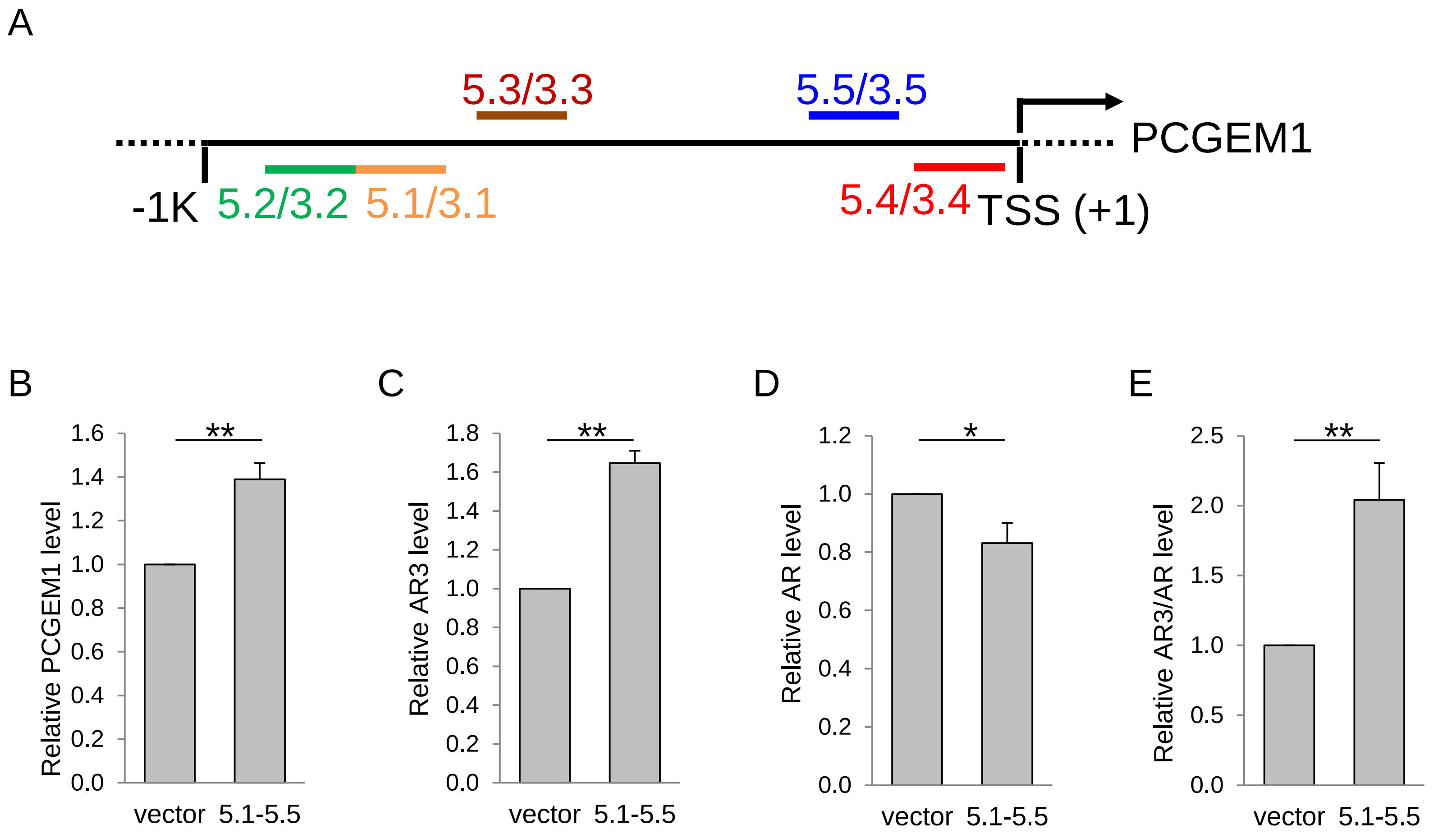

Fig. S6 SAM-mediated sgRNAs activate PCGEM1 and AR3. (A) Schematic presentation of PCGEM1 promoter, and the targeting locations of the respective SAM-mediated sgRNAs. The numbers below the PCGEM1 promoter indicate the position relative to TSS. Fold activation of (B) PCGEM1 and (C) AR3 transcripts by SAM (a combination of five sgRNAs). (D) A decrease in total AR expression by SAM. (E) An increase ratio of AR3 over AR by SAM. Values are mean ± SE (n = 3). *, $p < 0.05$. **, $p < 0.01$.

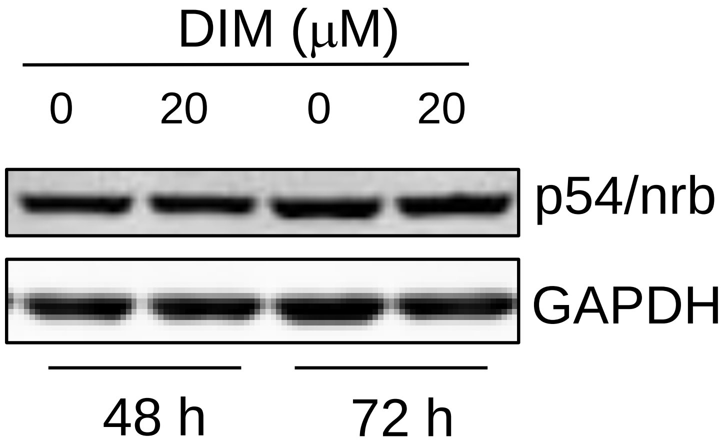

Fig. S7 DIM has no effect on the p54/nrb level. CWR22Rv1 cells were treated with vehicle control (DMSO) or 20 μM of DIM for 48 h and 72 h, and the p54/nrb protein levels were determined by Western blot.

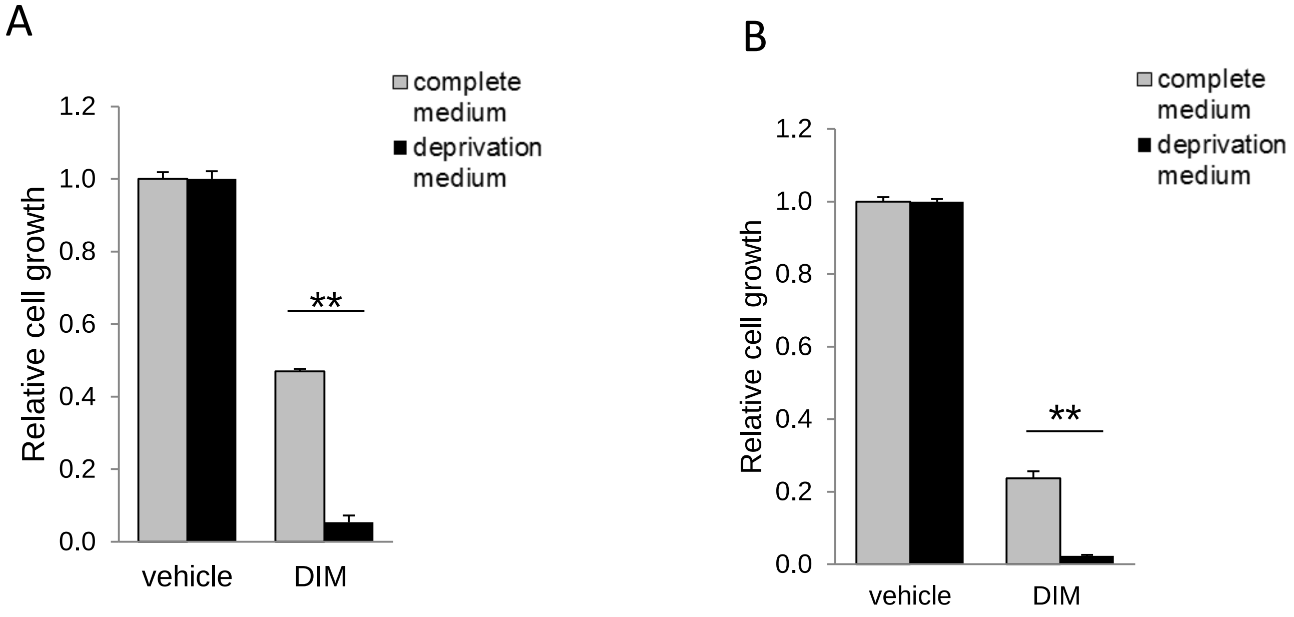

Fig. S8 DIM sensitizes LNCaP (A) and CWR22Rv1 (B) cells to androgen deprivation. Cells were plated out and cultured in 96-well plates in the presence (complete medium) or absence of androgen (deprivation medium). These cells were subject to vehicle control (DMSO) or 50 µM of DIM. Relative cell growth was measured by MTT assays at Day 4 after DIM administration. Values are mean ± SE (n = 3). **, $p < 0.01$.

Additional supplementary materials

Quantification of proteins associated with figures as indicated

1C

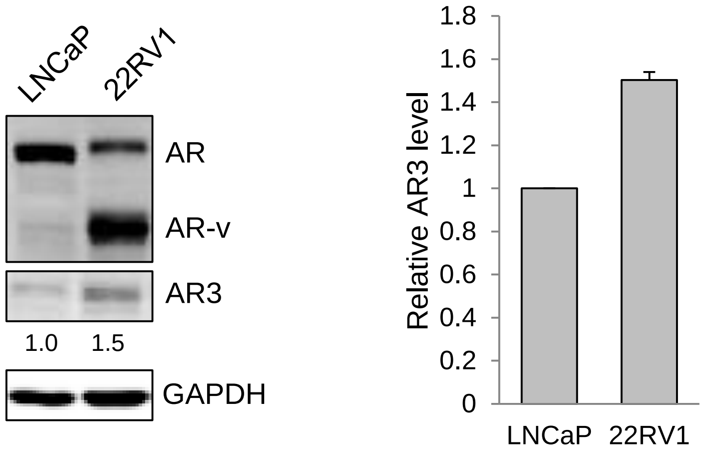

3C

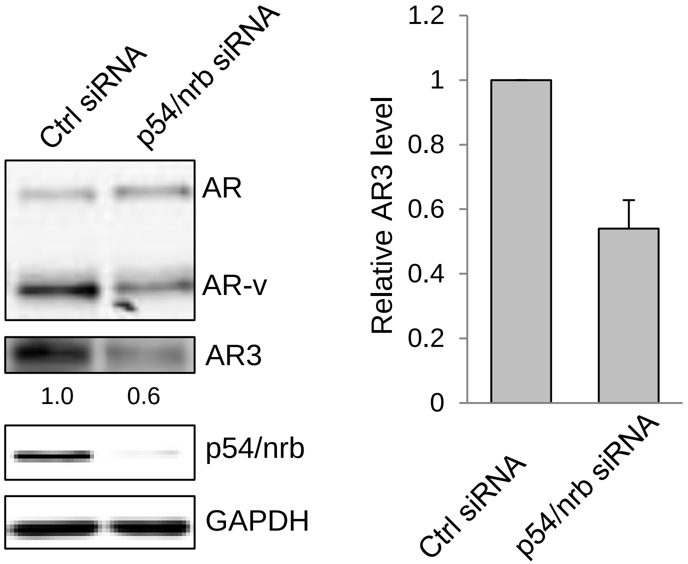

4B

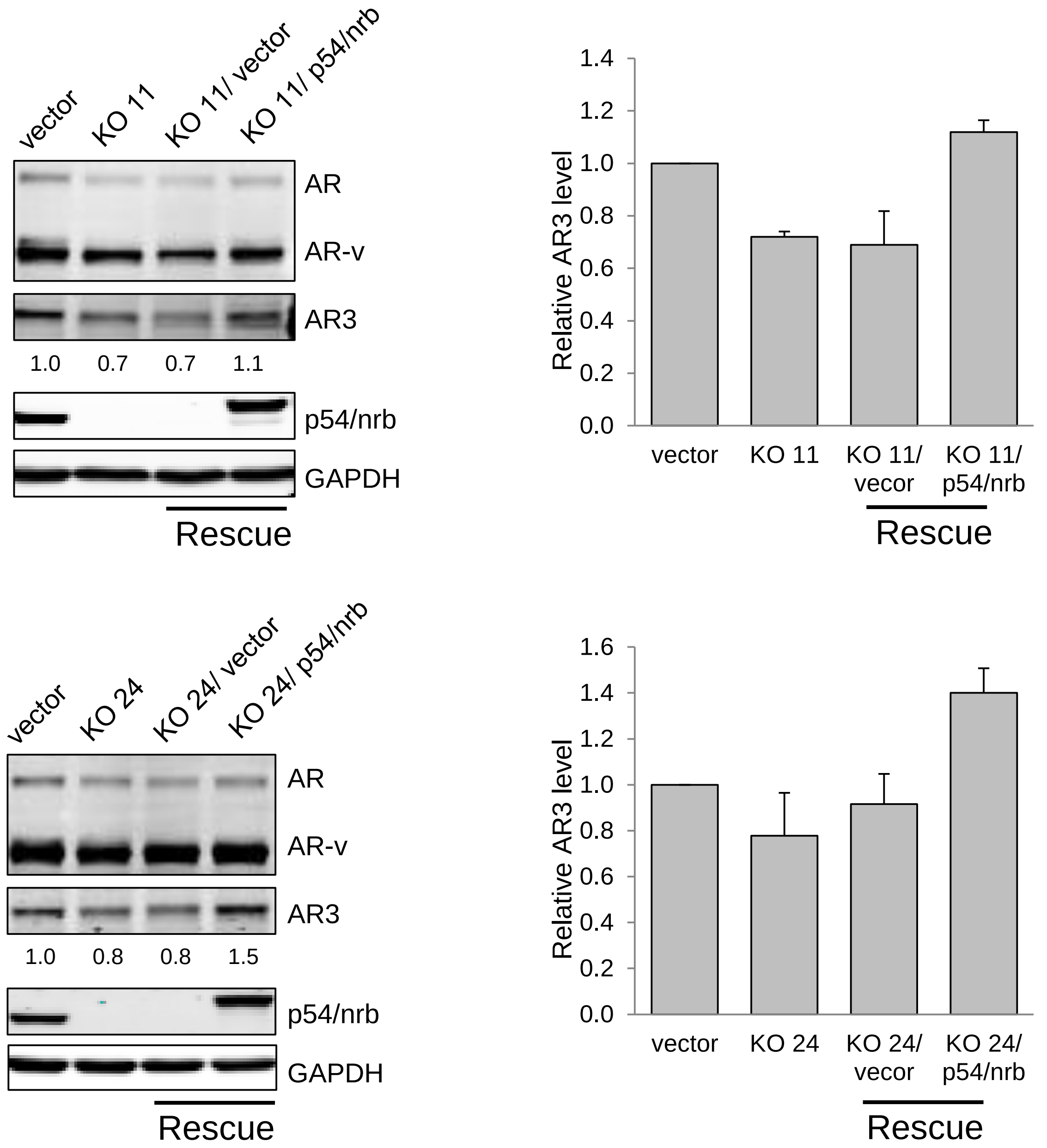
vector
KO 11
KO 11/ vector
KO 11/ p54/nrb
AR
AR-v
AR3
1.0 0.7 0.7 1.1
p54/nrb
GAPDH
Rescue
Relative AR3 level
1.4
1.2
1.0
0.8
0.6
0.4
0.2
0.0
vector
KO 11
KO 11/ vecor
KO 11/ p54/nrb
Rescue
vector
KO 24
KO 24/ vector
KO 24/ p54/nrb
AR
AR-v
AR3
1.0 0.8 0.8 1.5
p54/nrb
GAPDH
Rescue
Relative AR3 level
1.6
1.4
1.2
1.0
0.8
0.6
0.4
0.2
0.0
vector
KO 24
KO 24/ vecor
KO 24/ p54/nrb
Rescue

5B

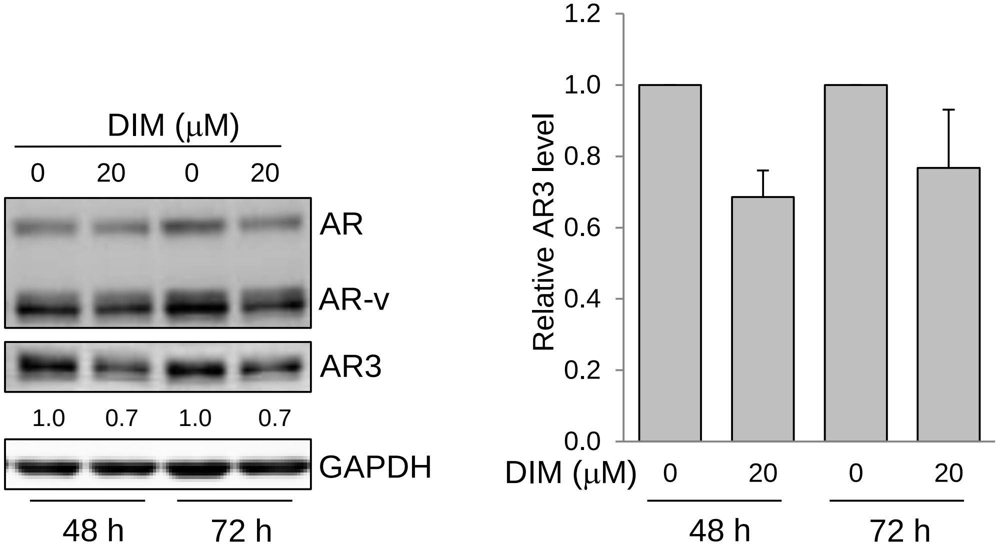
DIM (μM)
0
20
0
20
AR
AR-v
AR3
1.0
0.7
1.0
0.7
GAPDH
48 h
72 h
Relative AR3 level
1.2
1.0
0.8
0.6
0.4
0.2
0.0
DIM (μM)
0
20
0
20
48 h
72 h

Original gel pictures associated with figures indicated

Fig. 1C

1C

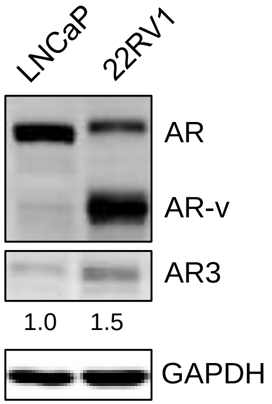

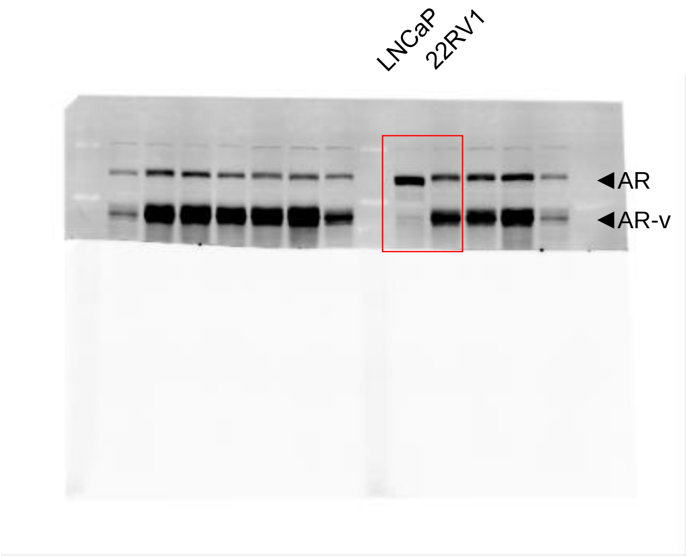

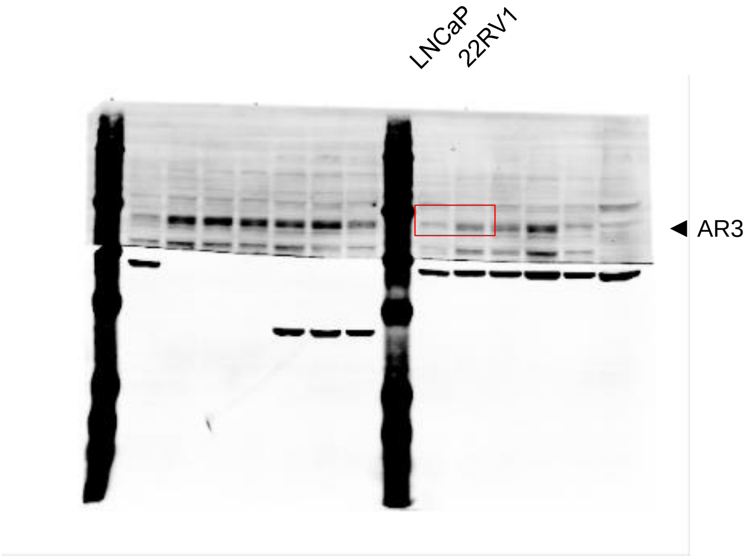

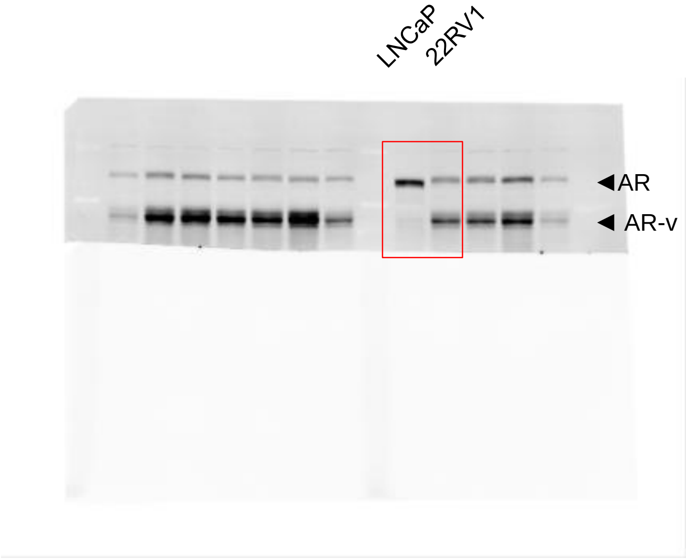

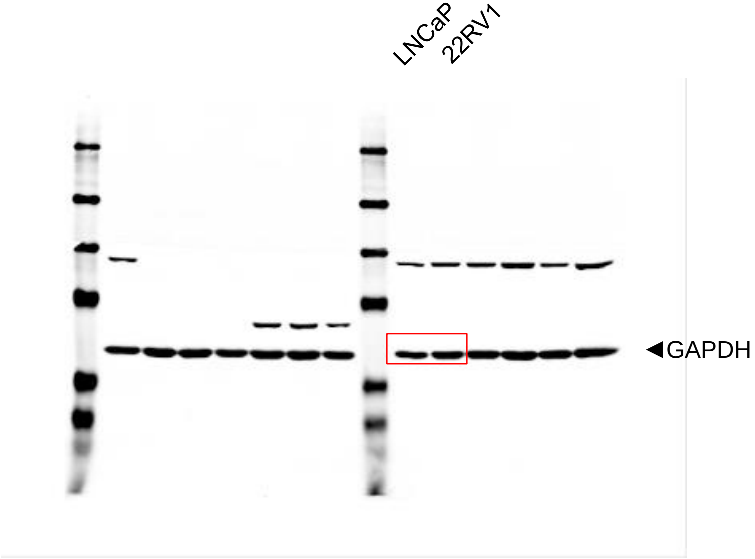

Fig. 2B

2B

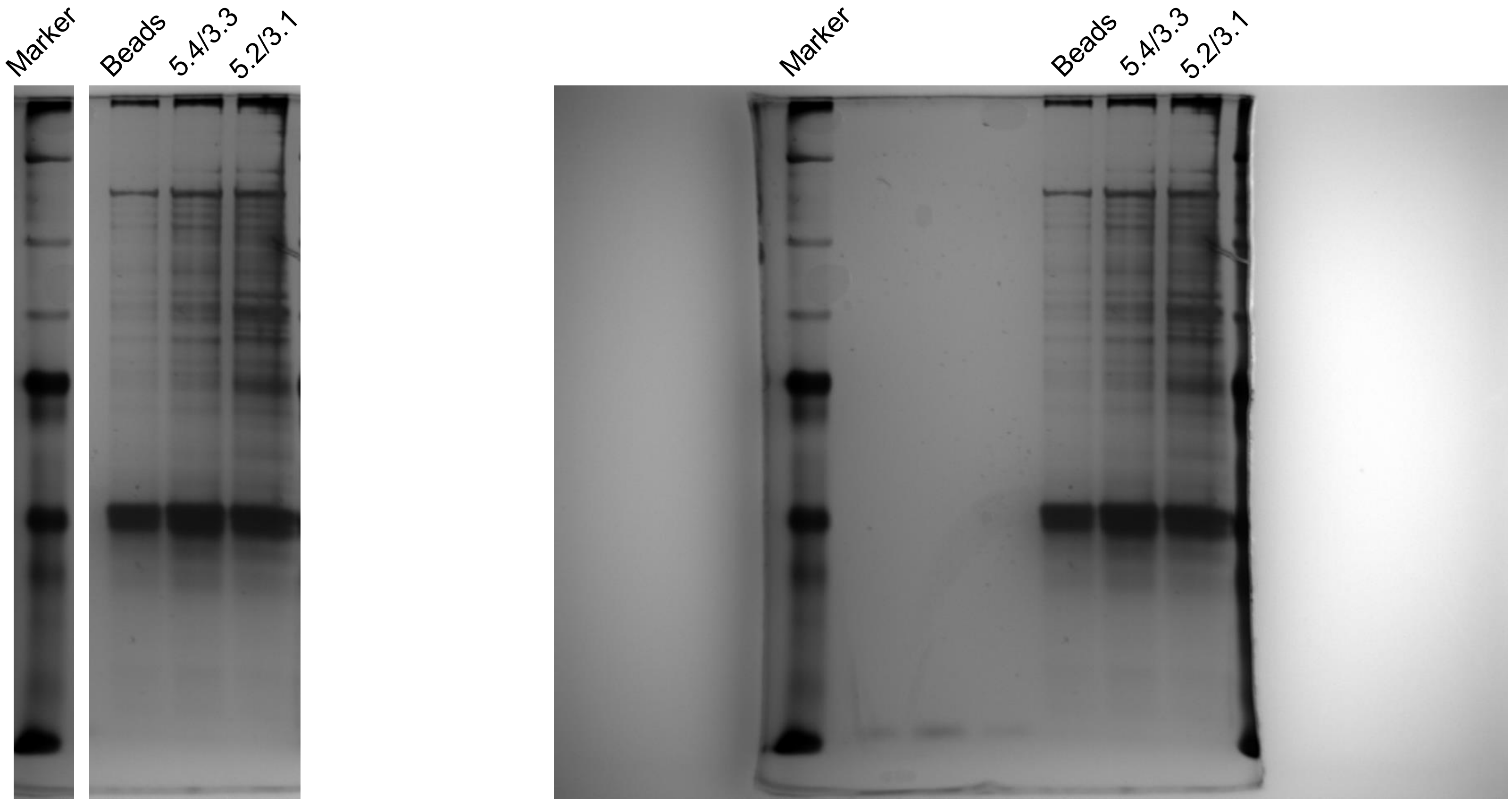

Fig. 2C

2C

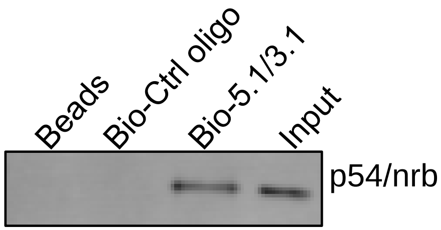

Pulldown & Western

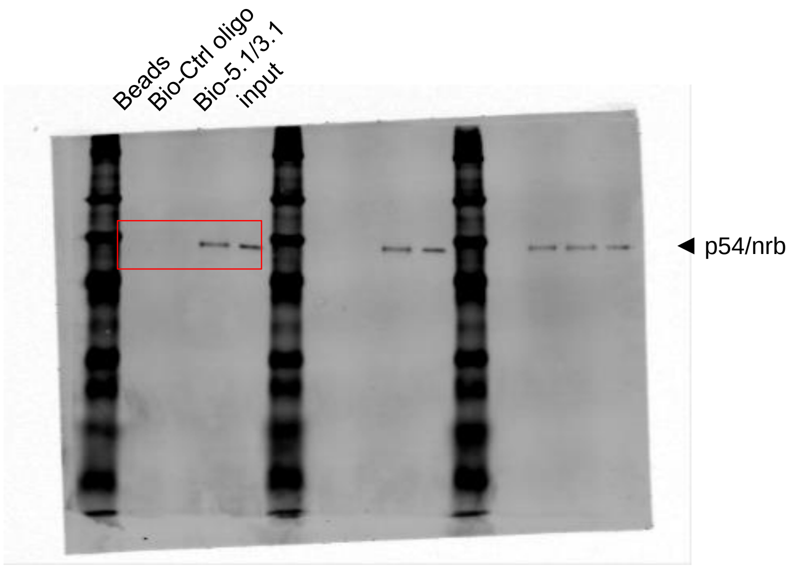

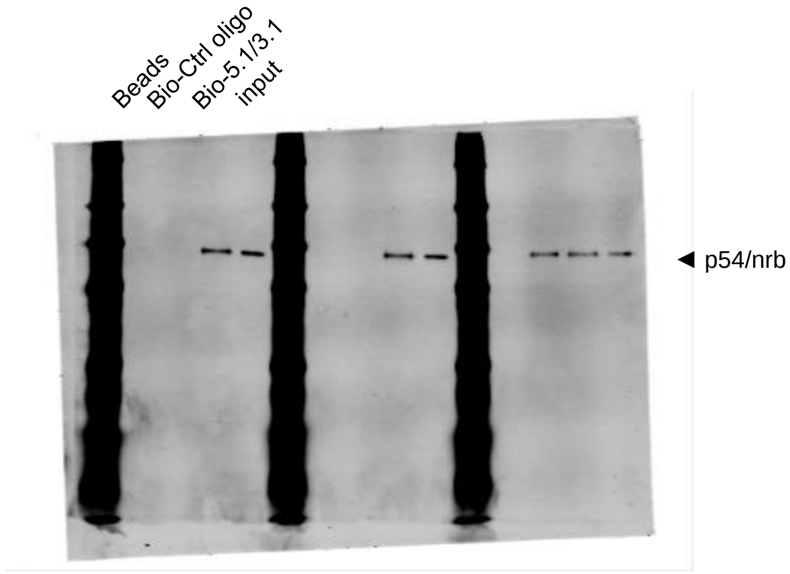

Fig. 2D

2D

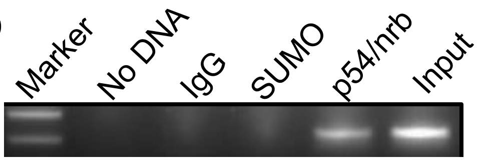

ChIP with primers ChiP-5.1/3.1

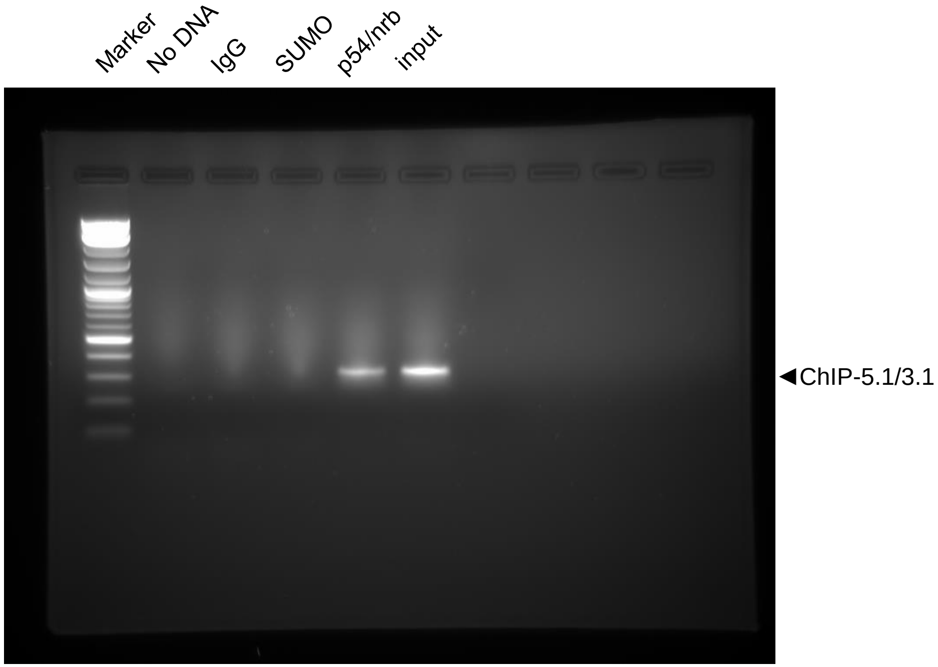

Fig. 3C

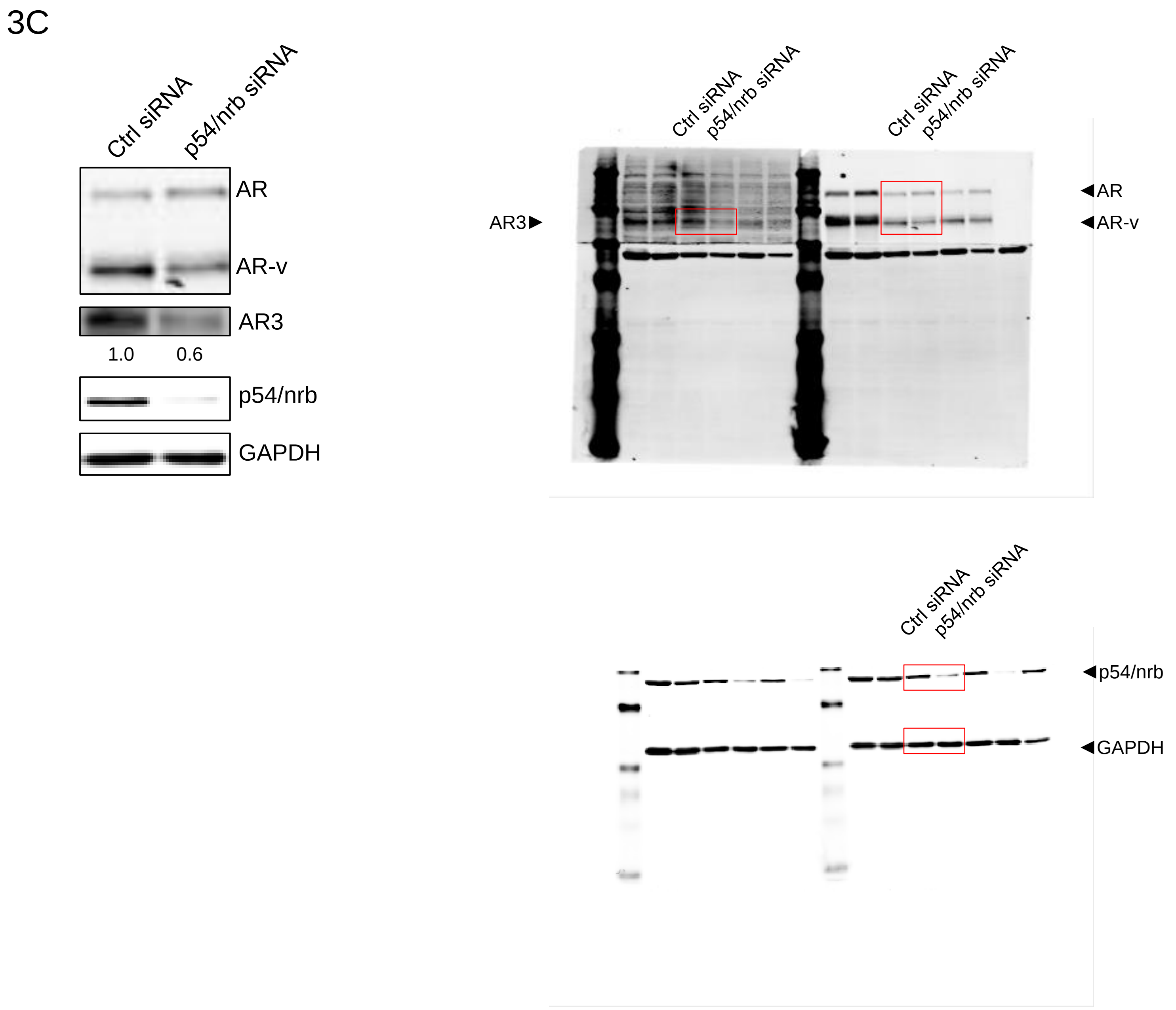
3C
Ctrl siRNA
p54/nrb siRNA
AR
AR-v
AR3
1.0
0.6
p54/nrb
GAPDH
Ctrl siRNA
p54/nrb siRNA
Ctrl siRNA
p54/nrb siRNA
AR3
AR
AR-v
Ctrl siRNA
p54/nrb siRNA
p54/nrb
GAPDH

Fig. 4B

4B

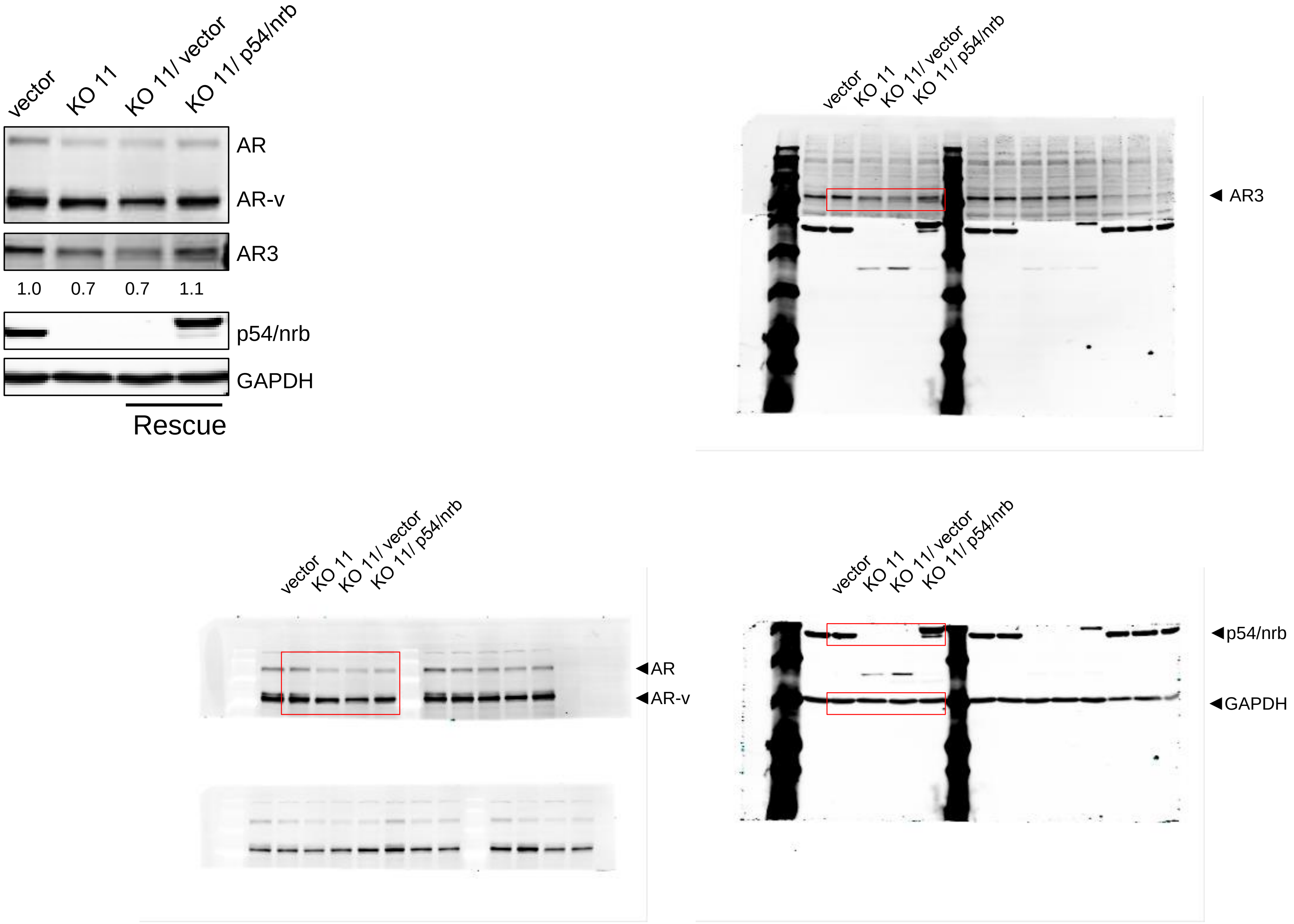
vector
KO 11
KO 11/ vector
KO 11/ p54/nrb
AR
AR-v
AR3
1.0
0.7
0.7
1.1
p54/nrb
GAPDH
Rescue
vector
KO 11
KO 11/ vector
KO 11/ p54/nrb
◀ AR3
vector
KO 11
KO 11/ vector
KO 11/ p54/nrb
◀AR
◀AR-v
vector
KO 11
KO 11/ vector
KO 11/ p54/nrb
◀p54/nrb
◀GAPDH

Fig. 4B *(cont'd)*

4B *(cont'd)*

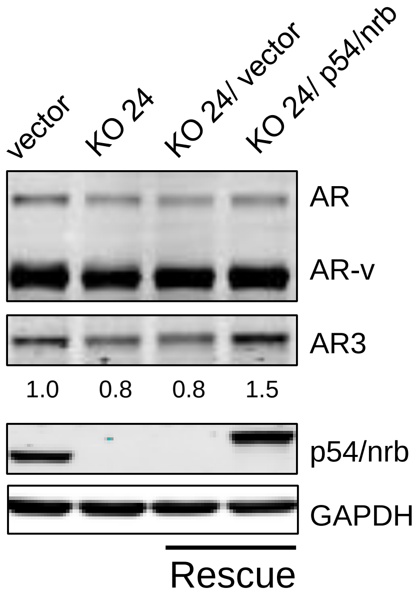

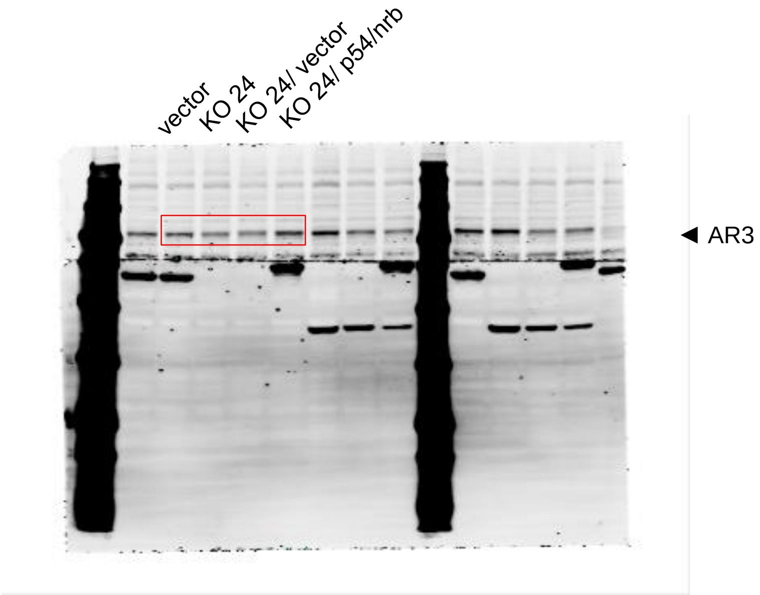

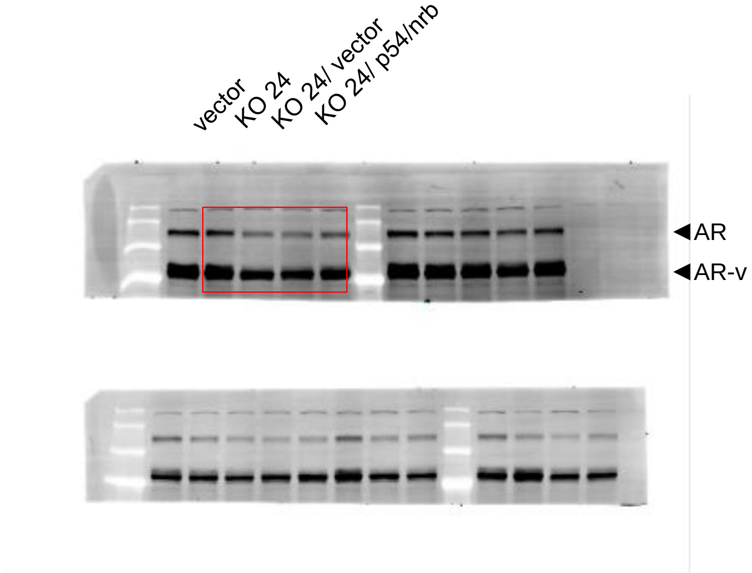

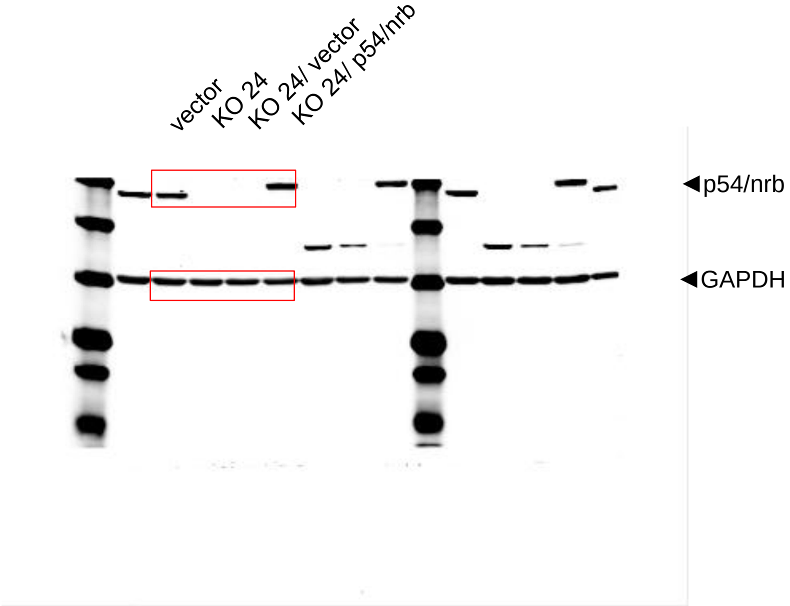

Fig. 5B

5B

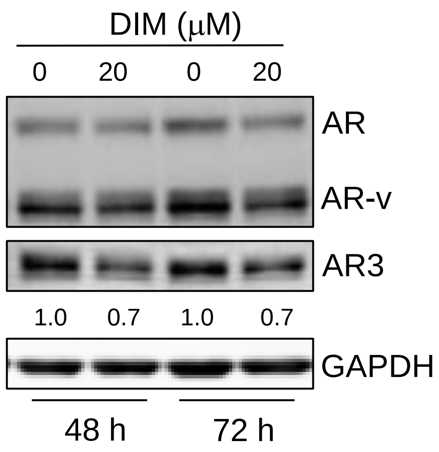
DIM (μM)
0 20 0 20
AR
AR-v
AR3
1.0 0.7 1.0 0.7
GAPDH
48 h
72 h

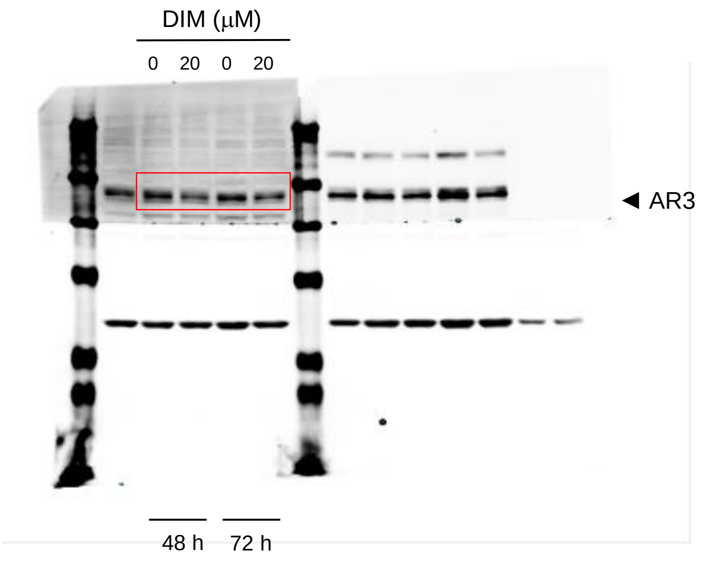
DIM (μM)
0 20 0 20
AR3
48 h
72 h

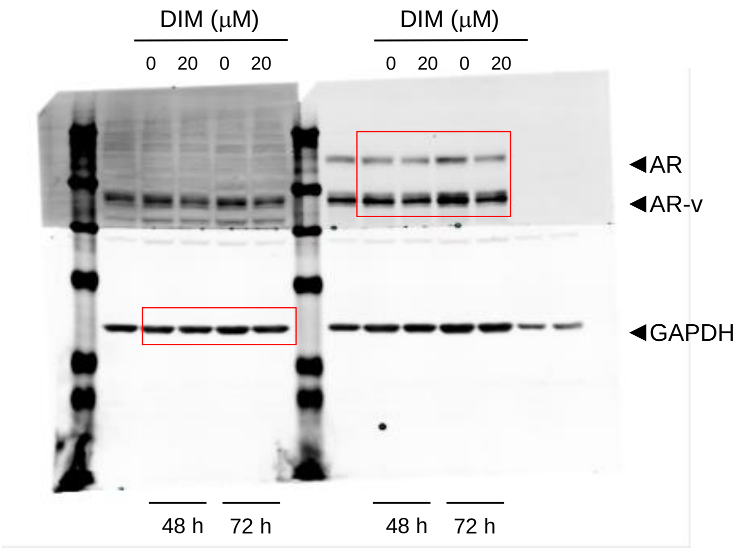
DIM (μM)
0 20 0 20
DIM (μM)
0 20 0 20
AR
AR-v
GAPDH
48 h
72 h
48 h
72 h

Fig. 5C

5C

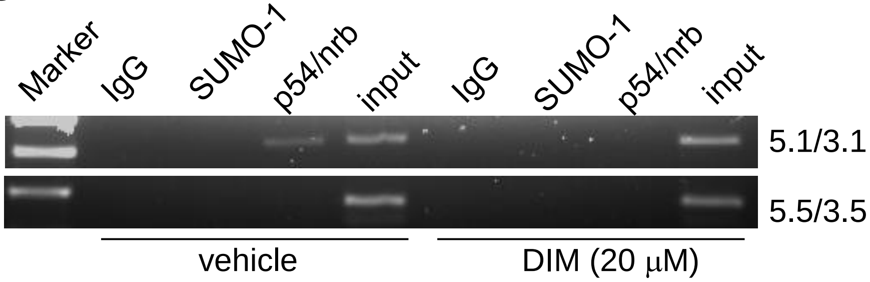
Marker
IgG
SUMO-1
p54/nrb
input
IgG
SUMO-1
p54/nrb
input
5.1/3.1
5.5/3.5
vehicle
DIM (20 μM)

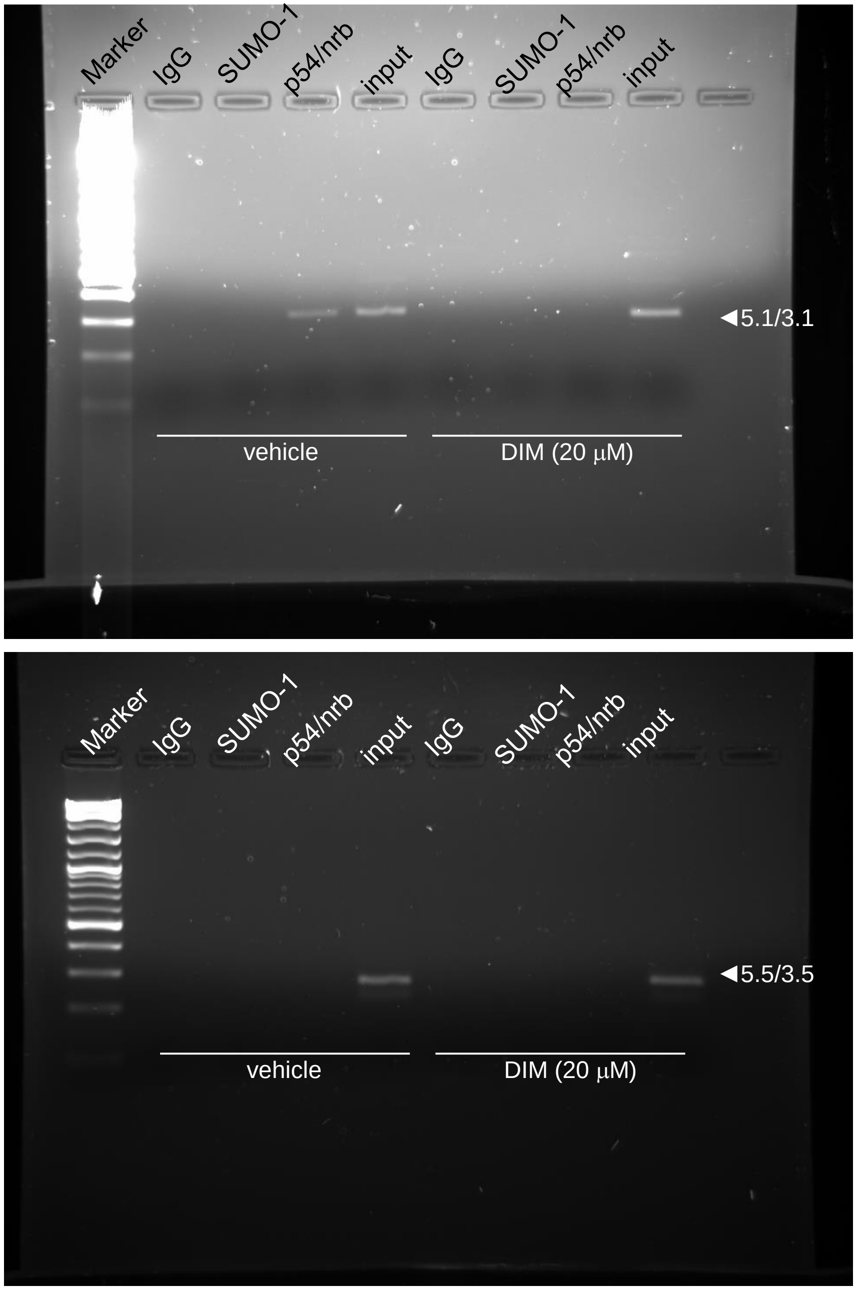
Marker
IgG
SUMO-1
p54/nrb
input
IgG
SUMO-1
p54/nrb
input
◄5.1/3.1
vehicle
DIM (20 μM)
Marker
IgG
SUMO-1
p54/nrb
input
IgG
SUMO-1
p54/nrb
input
◄5.5/3.5
vehicle
DIM (20 μM)

Fig. 5D

5D

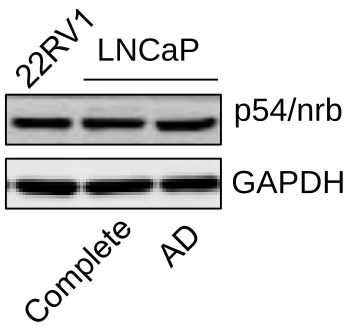
22RV1
LNCaP
p54/nrb
GAPDH
Complete
AD

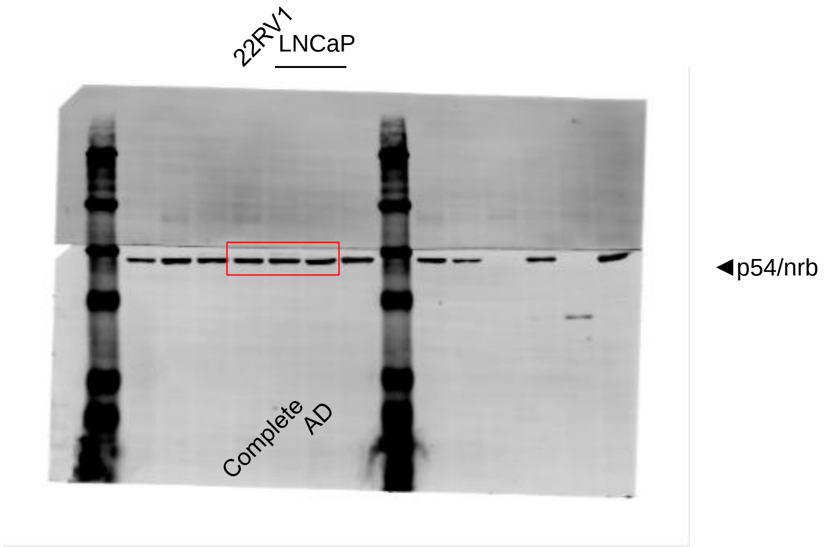
22RV1
LNCaP
Complete
AD
◀p54/nrb

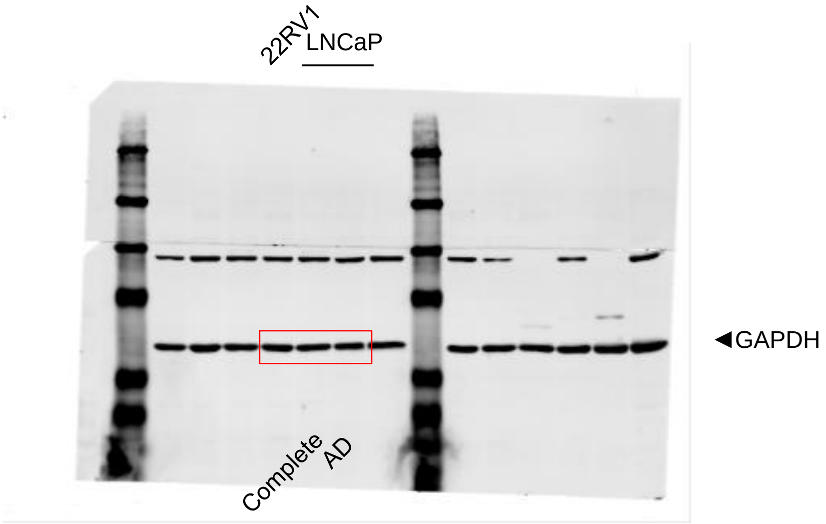
22RV1
LNCaP
Complete
AD
◀GAPDH

Fig. 5E

5E

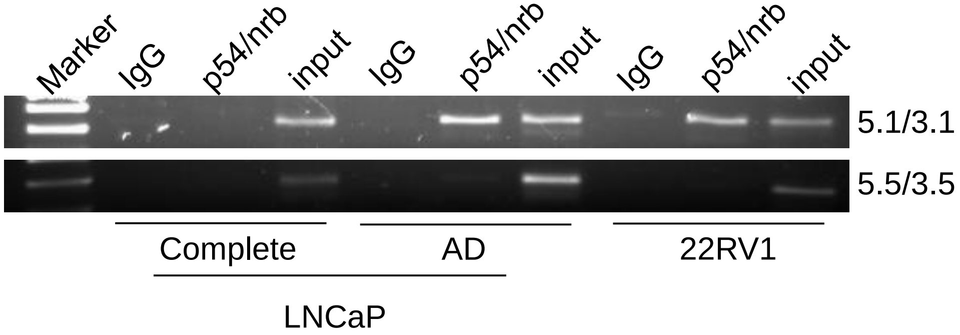
Marker
IgG
p54/nrb
input
IgG
p54/nrb
input
IgG
p54/nrb
input
5.1/3.1
5.5/3.5
Complete
AD
22RV1
LNCaP

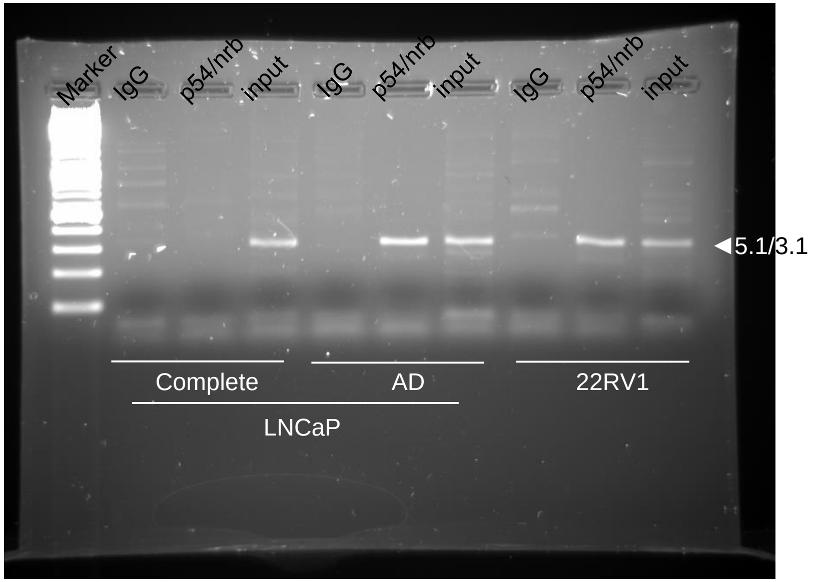
Marker
IgG
p54/nrb
input
IgG
p54/nrb
input
IgG
p54/nrb
input
5.1/3.1
Complete
AD
22RV1
LNCaP

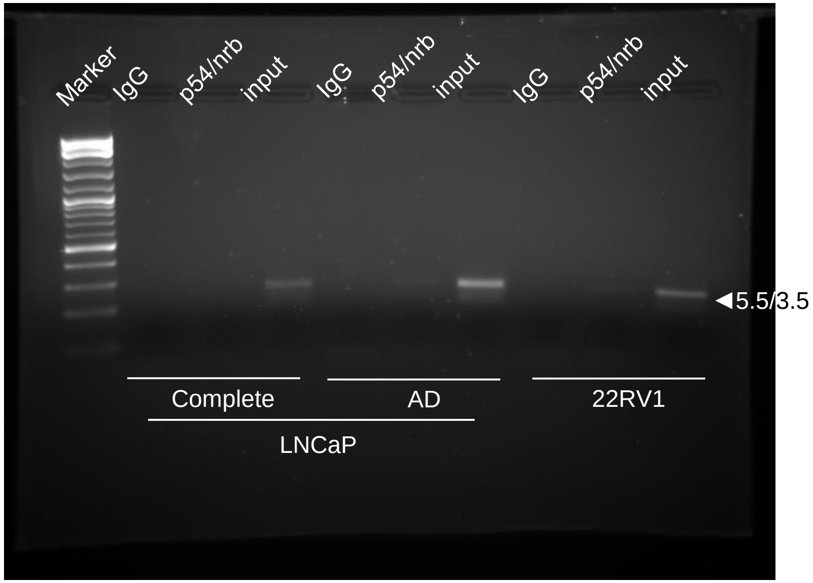
Marker
IgG
p54/nrb
input
IgG
p54/nrb
input
IgG
p54/nrb
input
5.5/3.5
Complete
AD
22RV1
LNCaP
